# H3K14ac is linked to methylation of H3K9 by the triple Tudor domain of SETDB1

**DOI:** 10.1038/s41467-017-02259-9

**Published:** 2017-12-12

**Authors:** Renata Z. Jurkowska, Su Qin, Goran Kungulovski, Wolfram Tempel, Yanli Liu, Pavel Bashtrykov, Judith Stiefelmaier, Tomasz P. Jurkowski, Srikanth Kudithipudi, Sara Weirich, Raluca Tamas, Hong Wu, Ludmila Dombrovski, Peter Loppnau, Richard Reinhardt, Jinrong Min, Albert Jeltsch

**Affiliations:** 1 Department of Biochemistry, Institute of Biochemistry and Technical Biochemistry, Stuttgart University, Allmandring 31, 70569 Stuttgart, Germany; 2 Structural Genomics Consortium, University of Toronto, 101 College Street, Toronto, ON M5G 1L7 Canada; 3 Life Science Research Center, Southern University of Science and Technology, 518055 Shenzhen, China; 4 Max-Planck-Genomzentrum Köln, Carl-von-Linné-Weg 10, 50829 Köln, Germany; 5 Department of Physiology, University of Toronto, Toronto, ON M5S 1A8 Canada

## Abstract

SETDB1 is an essential H3K9 methyltransferase involved in silencing of retroviruses and gene regulation. We show here that its triple Tudor domain (3TD) specifically binds to doubly modified histone H3 containing K14 acetylation and K9 methylation. Crystal structures of 3TD in complex with H3K14ac/K9me peptides reveal that peptide binding and K14ac recognition occurs at the interface between Tudor domains (TD) TD2 and TD3. Structural and biochemical data demonstrate a pocket switch mechanism in histone code reading, because K9me1 or K9me2 is preferentially recognized by the aromatic cage of TD3, while K9me3 selectively binds to TD2. Mutations in the K14ac/K9me binding sites change the sub-nuclear localization of 3TD. ChIP-seq analyses show that SETDB1 is enriched at H3K9me3 regions and K9me3/K14ac is enriched at SETDB1 binding sites overlapping with LINE elements, suggesting that recruitment of the SETDB1 complex to K14ac/K9me regions has a role in silencing of active genomic regions.

## Abstract (web summary)

SETDB1 is a histone methyltransferase that generates H3K9me3 marks in euchromatic regions. Here the authors show that the triple Tudor domain (3TD) of SETDB1 binds histone H3 tails containing K14 acetylation combined with K9 methylation, and that the K9me–K14ac modification defines a novel chromatin state enriched at SETDB1 binding sites.

## Introduction

Histone posttranslational modifications (PTMs) are essential for the regulation of chromatin states^1,2^. Trimethylation of histone H3 at lysine 9 (H3K9me3) is a hallmark of facultative and constitutive heterochromatin in almost all eukaryotes^3–6^, and it is also enriched in silenced genes^7^. In contrast, monomethylated H3K9 is found in the promoter regions of active genes^7^ and histone acetylation is a signal of active transcription and open chromatin^8^. SETDB1 (also known as ESET or KMT1E) is a histone H3K9 methyltransferase that generates H3K9me3 in euchromatic regions^9^. Gene knockout studies have shown that it is essential for early development in mice^10^. SETDB1 plays a critical role in gene regulation^11^, and it forms a complex with KAP-1 (also called TRIM28) that is required in embryonic stem cells^12,13^, primordial germ cells^14^, and B lymphocytes^15^. SETDB1 is also recruited to target sites by the HUSH complex^16^ and it associates with additional silencing factors including DNMT3A^17^, other H3K9 PKMTs^18^, and the NuRD HDAC chromatin remodeling complex^19–21^. The KAP-1/SETDB1 complex is required for silencing of LTR retroviruses^12–15^, and it has also been connected to H3K9 methylation of LINE elements^14,15,22–24^.

**Fig. 1:**
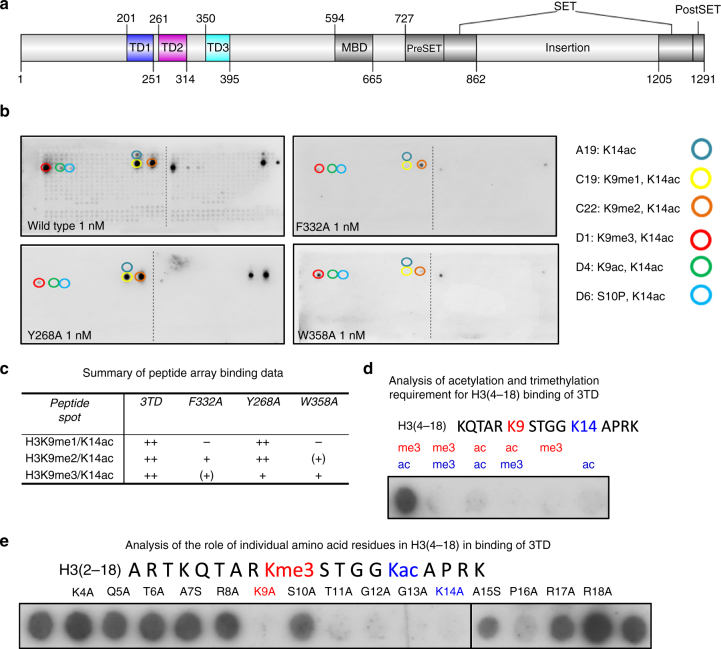
Peptide binding of SETDB1 Tudor domains. **a** Domain structure of SETDB1. **b** Modified histone peptide array binding of the 3TD and 3TD mutants. Peptide spots are annotated with the color-coded circles denoting the presence of the designated modifications. The full annotation of all peptide spots is provided in Supplementary Table 2. **c** Summary of the binding of 3TD and 3TD mutants to K9me1/2/3–K14ac peptides on peptide arrays shown in (**b**). **d** Peptide SPOT array binding of 3TD showing the double specificity of binding to K9me3 and K14ac. H3 (4–18) peptides were synthesized containing lysine trimethylation (me3) or acetylation (ac) at K9 and K14 and binding of 3TD analyzed. **e** Peptide SPOT array binding to map the recognition sequence of 3TD. The first and last spots contain the H3 (2–18) K9me3/K14ac peptide. The other spots contain peptides with the indicated amino acid exchanges. The data reveal that 3TD interacts with residues from K9 to P16

Histone-modifying enzymes often contain small functional domains for readout of specific histone PTMs that are involved in their recruitment and regulation of their enzymatic activity^25^. SETDB1 contains three Tudor domains (3TD) and a methyl-CpG binding domain (MBD) in its N-terminal part, followed by a split SET domain, which harbors the catalytic center for H3K9 methylation (Fig. 1a). Tudor domains are known to recognize methylated lysine and arginine^26,27^, but the potential binding ligand of the SETDB1 Tudor domains and their role in SETDB1 function remains unclear. We show here that SETDB1 3TD binds to histone H3 tails containing K14 acetylation combined with K9 methylation. Crystal structures revealed that K14ac recognition occurs at the interface between Tudor domain 2 (TD2) and Tudor domain 3 (TD3). Depending on the methylation state of K9, the methyllysine is preferentially bound by the aromatic cage of TD3 (K9me1 and K9me2) or TD2 (K9me3). Ectopic expression of 3TD wildtype and mutants fused to fluorophores demonstrates that the intact K14ac/K9me binding sites have an important role in the sub-nuclear localization of 3TD. We show that K9me3/K14ac regions bound by SETDB1 are enriched in LINE elements and propose that 3TD-mediated recruitment of SETDB1 to chromatin-containing active marks (K14ac) contributes to the efficient silencing of these regions. In summary, we characterize a reader for the combined K14ac/K9me mark, demonstrate the role of Tudor domains in binding acetyllysine, and identify a pocket switch mechanism in histone reading domains. Future work will show if this modification-dependent conformational change of the substrate triggers any downstream effects in SETDB1 or any of its complex partners.

## Results

### 3TD binds H3 peptides containing K9me1/2/3 and K14ac

**Fig. 2:**
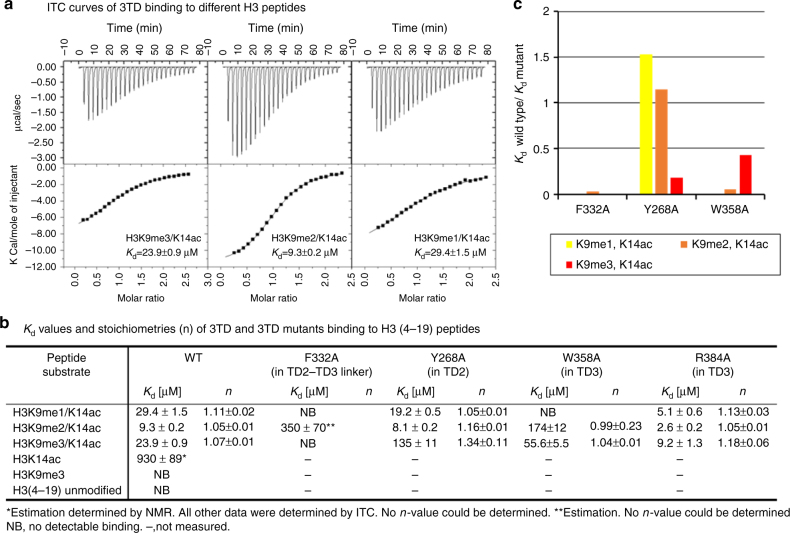
3TD binding to different H3 peptides analyzed by ITC. **a** Representative ITC curves and dissociation constants of 3TD binding to different H3 peptides. **b** Compilation of dissociation constants of 3TD and 3TD mutants to different peptides (Supplementary Figs. 1b,c, and 6). **c** Relative binding affinity of F332A, Y268A, and W358A to K9me1/2/3-K14ac peptides. While F332A has generally reduced binding, Y268A shows a specific loss in binding of K9me3–K14ac and W385A shows a preferential reduction of K9me1/2–K14ac binding

To study the function of the triple Tudor domain (3TD) of SETDB1, we first investigated the histone peptide binding ability of the GST-tagged 3TD. Using modified histone peptide arrays, we identified strong and selective binding of 3TD to three combinatorially modified H3 peptides: H3K9me1/K14ac, H3K9me2/K14ac, and H3K9me3/K14ac (Fig. 1b). Peptide array binding experiments at 100-fold increased concentration of 3TD detected weaker binding to monomodified H3K14ac, but no binding to H3K9me (Supplementary Fig. 1a). To further evaluate the relative contribution of each modification to the overall binding, we performed Isothermal Titration Calorimetry (ITC) and NMR chemical shift perturbation binding experiments with different peptides, which confirmed the binding and revealed dissociation constants (*K*_d_) in the lower micromolar range for H3K9me/K14ac peptides (Fig. 2a, b). In agreement with the peptide array binding results, these experiments revealed that although the acetylation at H3K14 is indispensable for 3TD binding, binding to monomodified H3K14ac was very weak. Methylation of H3K9 alone was insufficient for binding, but it strongly enhanced binding when combined with K14ac (Fig. 2b, c, Supplementary Fig. 1b, c). Peptide SPOT array binding experiments confirmed the requirement of K9me/K14ac for 3TD binding (Fig. 1d). In addition, binding of 3TD to an alanine scanning peptide SPOT array showed that the H3 sequence between T11 and P16 is critical for the interaction (Fig. 1e). The binding of the Tudor domains of SETDB1 to methylated histone tails has been investigated previously, but no binding was observed^28^. This could be explained by the fact that the specific K9me/K14ac combination of modifications was not tested in that study. Taken together, we conclude that the 3TD domain of SETDB1 specifically recognizes the acetylation mark of H3K14 and methylation of H3K9 on the same histone tail strongly enhances the binding affinity.

### Mode of histone H3 binding by the 3TD domain of SETDB1

**Fig. 3:**
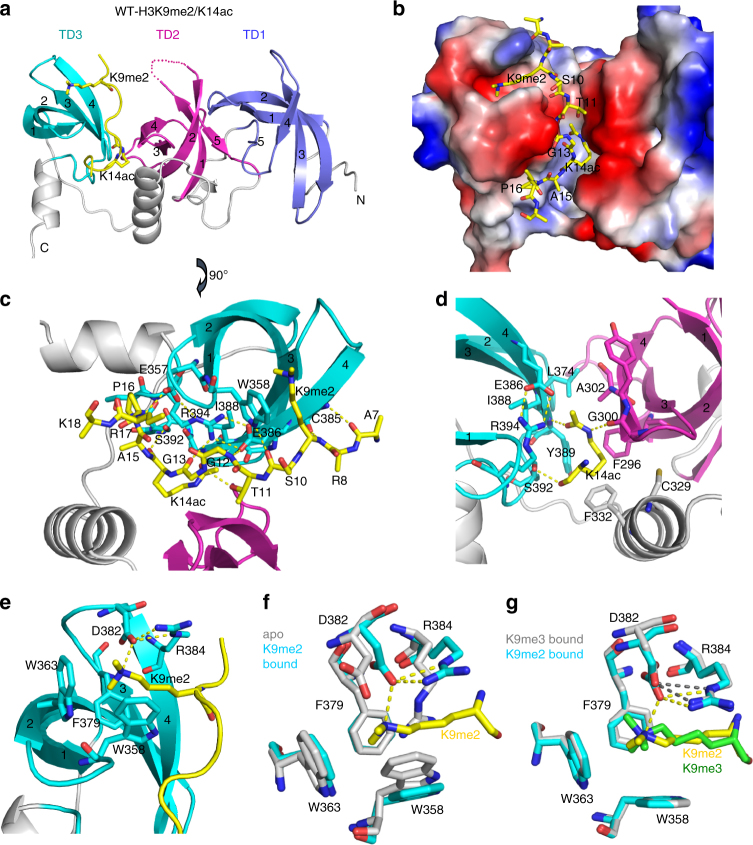
Details of the 3TD structure bound with a doubly modified peptide. **a** Structure of wildtype 3TD bound to H3K9me2/K14ac. 3TD is shown in ribbon representation with the peptide shown in yellow. **b** Surface representation of the protein in the same orientation as in (**a**) with the peptide shown in stick mode. **c** Details of the K9me2/K14ac peptide interaction in the cleft between TD2 and TD3. **d** Details of the K14ac recognition at the interface of TD2 and TD3. **e** Details of the K9me2-binding aromatic pocket in TD3. **f** Comparison of the aromatic pocket in TD3 in the apo form and **g** after binding of different H3K9me/K14ac peptides

To understand the molecular basis for the recognition of the combinatorial H3K9me/K14ac histone mark, we solved the crystal structure of 3TD in complex with an H3(4–19) K9me2/K14ac doubly modified peptide (Fig. 3, Supplementary Figs. 2 and 3, Supplementary Table 1). The three Tudor domains compose a stack, in which the individual domains interact with each other face-to-back. Each domain forms a β-barrel-like fold characteristic of Tudor domains^26^, with the barrel bottoms pointing in the same direction. Notably, there are extensive interactions among the Tudor domains and the inter-domain linkers, indicating that 3TD folds and functions as a single unit (Supplementary Fig. 2a). The histone H3 peptide lies in a groove formed by TD2, TD3, and the linker between them (Fig. 3a, b). The specificity of the H3 peptide interaction is determined mainly through (1) recognition of K14ac by an acetyl-lysine binding groove between TD2 and TD3, (2) recognition of K9me2 by an aromatic cage in TD3, and (3) binding of G12 and G13 to a narrow groove between TD2 and TD3. In agreement with this observation, peptide SPOT binding experiments showed that alanine residues at the positions of G12 and G13 of H3 disrupted the interaction (Fig. 1e), indicating that any bigger residue at this position would preclude peptide binding. Moreover, E386 and R394 form a salt bridge inside TD3 and the backbone of G12–G13 is hydrogen-bonded to them (Fig. 3c). Binding to modified histone peptide arrays showed that mutation of E386 or R394 abolished the peptide-binding ability of the Tudor domains (Supplementary Fig. 4a).

The K14ac is recognized by residues from TD2, TD3, and the linker between them (Fig. 3d). The acetyl group forms two hydrogen bonds with the backbone carbonyl-oxygen of G300 and the backbone amine of Y389. The side chains of F332, F296, A302, and Y389 form hydrophobic interactions with the acetyl-lysine. Replacement of F332 by alanine caused a strong reduction in affinity of 3TD to the H3K9me2/K14ac peptide in peptide array and ITC peptide-binding experiments (Figs. 1 and 2, Supplementary Fig. 4a), and mutation of I388, which is a part of the hydrophobic core of TD3, disrupted the binding completely (Supplementary Fig. 4a), confirming the importance of these hydrophobic interactions. In addition, the backbone of the histone peptide forms a series of hydrogen bonds with the residues from TD3 further stabilizing the complex (Fig. 3c).

### TD3 preferentially recognizes dimethylated K9

The K9me2 side chain is inserted into an aromatic cage of TD3 formed by W358, W363, and F379 (Fig. 3e). A direct hydrogen bond between the carboxylate of D382 and the ε-ammonium of K9me2 appears to further stabilize the complex explaining the preference of this aromatic cage for binding of K9me2 over K9me3. Disruption of the aromatic cage by mutation of W358 or W363 abrogated H3K9me1 binding and reduced H3K9me2 peptide binding in peptide array binding (Fig. 1 and Supplementary Fig. 4d) and ITC peptide-binding experiments (Fig. 2, Supplementary Fig. 6c), while H3K9me3 binding was only 2-fold reduced (see below). Similarly, mutation of D382 to alanine or arginine mainly reduced binding of K9me1 to the pocket, but did not affect the binding of K9me2 or K9me3 (Supplementary Fig. 4d). This can be explained by the fact that K9me1 has the weakest interactions with the aromatic cage, so that the D382-mediated hydrogen bond is more critical for K9me1 than for K9me2 binding (for K9me3 binding, also see below). Comparison with the apo-structure of 3TD revealed a conformational change associated with the methyl-lysine binding. In the absence of the bound peptide, R384 is packed into the aromatic cage, blocking the peptide-binding site (Fig. 3f). Consequently, alanine mutation of R384 enhanced the peptide-binding affinity of 3TD by 3- to 6-fold (Fig. 2b, Supplementary Fig. 6b).

We also solved the structures of 3TD in complex with H3K9me3/K14ac or H3K9me0/K14ac (Supplementary Fig. 3, and Supplementary Table 1). In the H3K9me3/K14ac bound structure, the H3 peptide interacts with 3TD in a similar mode as in the H3K9me2/K14ac complex. The K9me3 is inserted into the aromatic cage of TD3, and only slight rearrangements were observed in the K9–aromatic cage interaction (Fig. 3g). In the H3K9me0/K14ac complex structure, the unmethylated K9 and residues preceding it cannot be tracked clearly suggesting that they are disordered (Supplementary Fig. 3).

### TD1 contains an inactive and incomplete aromatic cage

Having shown that TD3 preferentially binds K9me2, we next asked whether TD1 and TD2 also have potential roles in histone binding. Sequence and structure alignments revealed that TD1 has an incomplete aromatic cage consisting of only H214 and F234. The position of the first aromatic residue is occupied by K208, suggesting that TD1 is unable to bind methylated lysine residues (Supplementary Fig. 2b and c). This was confirmed by a mutational study, as substitution of residues in TD1 did not or only weakly affected peptide binding (Supplementary Fig. 4b).

### TD2 preferentially recognizes trimethylated K9

TD2 has all conserved aromatic residues (Y268, W275, Y277, F297, and Y301), which are positioned proximal to the H3K9me/K14ac peptides mentioned above. To test whether TD2 contributes to peptide binding, we generated a Y268A mutant, which disrupts the aromatic cage in TD2, and used it for both peptide array binding and ITC peptide-binding assays. Interestingly, the Y268A mutant showed a clear reduction in affinity to the H3K9me3/K14ac peptide, while the affinities to H3K9me2/K14ac and H3K9me1/K14ac peptides were not affected (Fig. 1b, c, and Supplementary Fig. 4c). These data suggest that TD2 is involved in H3K9me3/K14ac peptide binding.

In contrast, the mutation of W358 in TD3 (W358A), an aromatic cage residue of TD3, led to a loss of H3K9me1/K14ac binding and a strong reduction in binding of H3K9me2/K14ac, while binding of H3K9me3/K14ac was only moderately reduced in the peptide array binding (Fig. 1b, c). In the ITC peptide-binding experiments, similar results were obtained; the affinity to H3K9me2/K14ac was reduced 18-fold and binding to H3K9me1/K14ac was completely lost, but H3K9me3/K14ac binding was reduced only mildly (2-fold) (Fig. 2 and Supplementary Fig. 6c). These data suggest that TD3 is mainly involved in H3K9me1/2/K14ac binding. The weaker binding of H3K9me1/2/K14ac to W358A could be explained by the loss of the H-bond between the W358 side chain and the carbonyl-oxygen of S10, leading to a general reduction in peptide binding. We conclude that both TD2 and TD3 contribute to K9me binding, with TD2 showing preference for H3K9me3 and TD3 preferring H3K9me1/2.

**Fig. 4:**
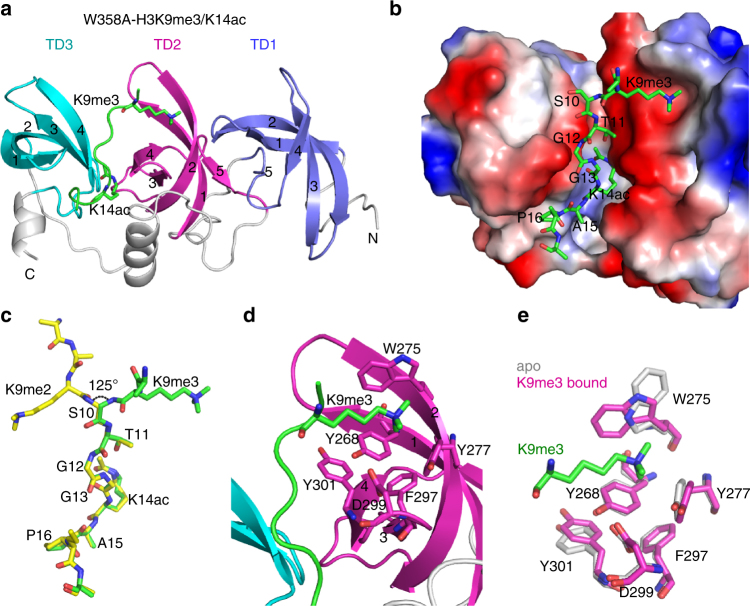
Details of the 3TD W358A structure bound with a doubly modified peptide. **a** Structure of 3TD W358A mutant bound to H3K9me3/K14ac shown in ribbon representation with the peptide shown in green. **b** Surface representation of the mutant in the same orientation as in (**a**). **c** Comparison of the conformations of the H3K9me2/K14ac (in yellow) and H3K9me3/K14ac (in green) peptides bound to wildtype 3TD and 3TD W358A mutant, respectively. The side chain of H3S10 is not shown for clarity. **d** Details of the K9me3-binding aromatic pocket in TD2. **e** Comparison of the aromatic pocket in TD2 in the apo form and after binding of the H3K9me3/K14ac peptides

We further solved the structures of the W358A mutant in complex with H3K9me3/K14ac or H3K9me2/K14ac peptides (Fig. 4 and Supplementary Table 1). In the W358A H3K9me3/K14ac structure, K9me3 indeed was bound to TD2, while the peptide residues following S10 (including K14ac) adopted the same conformation as in the wildtype structures (Fig. 4a–c). The K9me3 was bound to an open aromatic cage in TD2 formed by Y268, W275, and Y277, where W275 was rearranged compared to the apo-structure to accept the trimethylated K9 (Fig. 4d, e). In the W358A–H3K9me2/K14ac structure, electron density for the K9me2 residue did not permit confident placement of the dimethylated lysine, suggesting that it is partially disordered (Supplementary Fig. 3). In summary, the Tudor domains of SETDB1 collaborate to recognize the combinatorial histone marks H3K9me/K14ac, with distinct Tudor domains showing different methylation state selectivity.

### SETDB1 is active on K14ac substrates on peptide arrays

To investigate the peptide methylation activity of SETDB1, peptide arrays were methylated by the N-terminal-deleted SETDB1 protein (190–1291) (Supplementary Fig. 7). The results showed that SETDB1 methylates peptides containing K9 in un-, mono-, or dimethylated forms, indicating that SETDB1 could methylate histone H3K9 up to trimethylation. SETDB1 activity was observed on K14 acetylated and unacetylated substrates, and our data revealed a strong inhibition of the SETDB1 activity by phosphorylation of H3 peptides at S10 (as observed before^9^) or T11 (not yet reported). Previously, methylation experiments with peptides in solution showed that the K14ac modification inhibits the enzymatic activity of SETDB1^9^. The discrepancy of these results is possibly due to the different substrates used, because in solution, the SET and 3TD domains would compete for binding of substrate peptides, and acetylation of K14, which increases 3TD binding, would reduce catalytic activity. In contrast, in peptide array methylation experiments, the substrate peptides are present on the array at high density, and the binding of the 3TD to one peptide in a spot would allow the SET domain to methylate adjacent peptides in the same spot. Moreover, it cannot be ruled out that the different enzyme sources in both studies affected the results, because previously FLAG-tagged full-length SETDB1 purified from HEK293 cells was used, whereas we used an N-terminal-truncated SETDB1 purified from Sf9 cells. In the peptide array methylation experiments, peptides containing K14ac were methylated more strongly by SETDB1 (Supplementary Fig. 7), which would be in agreement with the recruitment of this enzyme to these peptide spots via the 3TD domain. However, given the limitations of the peptide array-based activity assay, this result needs further validation.

### H3 and nucleosomes binding of 3TD is K9me/K14ac dependent

**Fig. 5:**
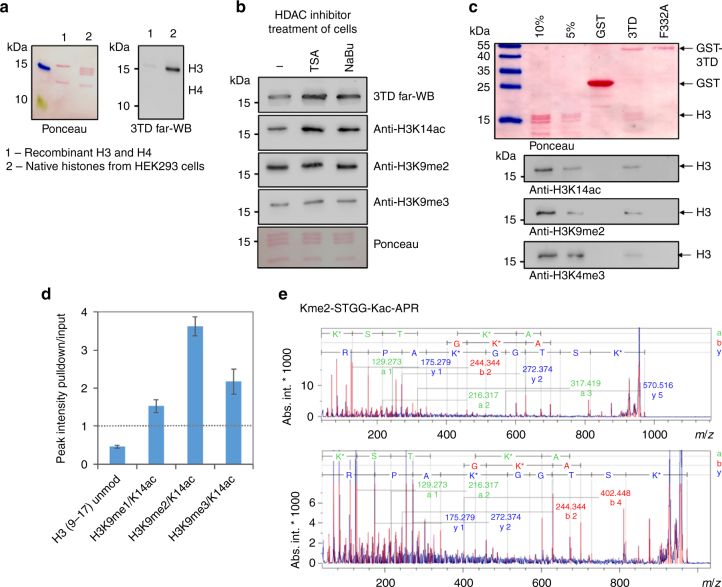
Binding of 3TD to modified H3 and nucleosomes. **a** Far-western blot analysis of 3TD binding to recombinant histones H3 and H4 (lane 1) and native histones (lane 2). Positions of the H3 and H4 proteins are indicated. The Ponceau-stained membrane is shown as a loading control. **b** Far-western blot analysis of 3TD binding to native histones isolated from HEK293 cells after treatment with histone deacetylase inhibitors trichostatin A (TSA) or sodium butyrate (NaBu). Untreated (−) cells are used as a control. The abundance of histone PTMs in the histone preparations was analyzed by western blot. The Ponceau-stained membrane is shown as a loading control. **c** Western blot analysis of the GST-3TD wildtype and F332A mutant mononucleosomal pull-down. Mononucleosomes were isolated from HEK293 cells. GST was used as a negative control. **d** MALDI MS analysis of the 3TD mononucleosomal pull-down. Quantification of the MS data (Supplementary Fig. 8) shows an enrichment of the H3K9me/K14ac peptides in 3TD pull-down and depletion of the unmodified H3(9–17) peptide when compared to input. Internal normalization was done using peaks of unrelated, unmodified H3 peptides. The error bars represent the SEM of two biological repeats. MS/MS for H3(9–17) and H3(41–49) are shown in Supplementary Fig. 8c,d. **e** MS/MS analysis of the H3(9–17) Kme2-STGG-Kac-APR peptide fragment (theoretical mass MH^+^: 971.563 Da: experimental mass: 971.482 Da). Fragment ions of the a, b, and y series are indicated in green, red, and blue, respectively. The two panels show the same analysis with different *x*- and *y*-axis scales

Having shown the specific binding of 3TD to H3 peptides modified with K14ac and K9me, we next aimed to investigate the role of H3 modifications in 3TD binding to native histones and nucleosomes. Native histones purified from human HEK293 cells and recombinant unmodified H3 were separated on SDS gels, and the binding of 3TD to the blotted histone proteins was investigated by far-western assays. As shown in Fig. 5a, 3TD specifically binds to the native histone H3 (containing endogenous modifications), but not to the unmodified recombinant H3. To investigate the role of lysine acetylation in 3TD binding to H3, we next used histones isolated from HEK293 cells treated with HDAC inhibitors (trichostatin A or sodium butyrate). Histones were separated on SDS gels and their modification state was analyzed by western blot with anti-K9me and anti-K14ac antibodies. As expected, H3 proteins isolated from cells treated with HDAC inhibitors showed an increased H3K14ac signal, while the content of K9 methylation did not change (Fig. 5b). Far-western blots were conducted to study 3TD binding to both histone preparations. Consistent with the gain in the H3K14ac level, an increased 3TD binding was observed, confirming that the 3TD binding to native histones is conferred by histone acetylation. Next, we used the GST-tagged 3TD to pull-down mononucleosomes in chromatin domain immunoprecipitation (CIDOP) experiments^29,30^. Wildtype 3TD, but not the F332A mutant (with disrupted acetyl-lysine binding pocket), was able to pull-down H3 containing K14ac and K9me2 (Fig. 5c). H3K4me3 was analyzed as unrelated active mark and found not to be enriched in the pull-down. The pull-down was analyzed by mass spectrometry and showed strong enrichment of doubly modified H3K9me/K14ac peptides (1.5 to 2-fold for K9me1/K14ac and K9me3/K14ac, up to 4-fold for K9me2/K14ac) (Fig. 5d and Supplementary Fig. 8). The identity of the K9me2–K14ac peptide was confirmed by MS/MS (Fig. 5e). Unmodified H3 (9–17) and unrelated peptides detected by mass spectrometry analysis were depleted or not enriched confirming the specificity of the pull-down reaction (Supplementary Fig. 8).

### SETDB1 co-localizes with H3K14ac/K9me3 chromatin

**Fig. 6:**
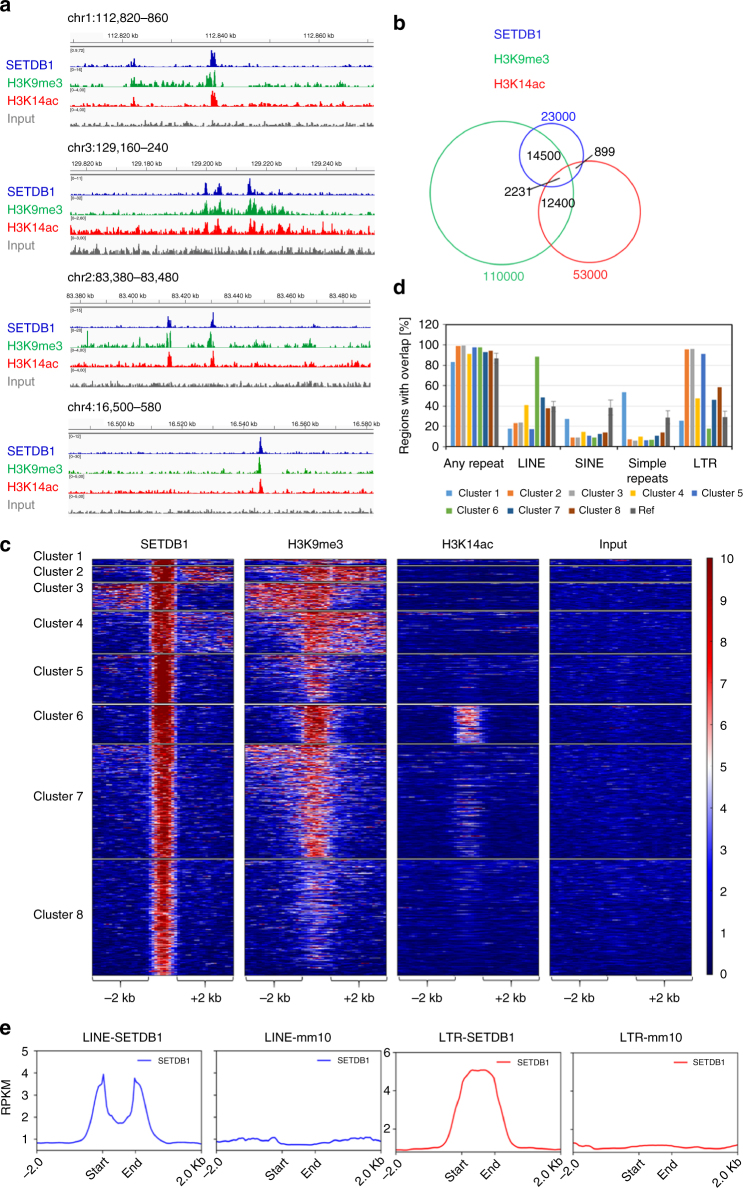
Genomic binding of SETDB1. **a** Example browser views of the co-occurrence of SETDB1, H3K9me3, and H3K14ac peaks. **b** Venn diagram of the overlap of annotated SETDB1, H3K9me3, and H3K14ac peaks. **c** Clustering analysis of RPKM read density centered on SETDB1 peaks (±2 kb) showing the strong overlap of SETDB1 with H3K9me3 in cluster 1–7 and the overlap of SETDB1, H3K9me3, and H3K14ac peaks in cluster 6. **d** Overlap of the SETDB1 binding regions from cluster 1–8 with different classes of repeat elements. “Ref” refers to the average of 10 control distributions calculated by EpiExplorer^61^ for the different clusters. The standard errors of the average repeat contents in these control distributions are indicated by the error bars. More details and statistics are provided in Supplementary Note 3. Note the strong enrichment of LTRs in clusters 2, 3, and 5 and of LINEs in cluster 6. **e** Meta-profiles of SETDB1 density on SETDB1 overlapping LINE (LINE-SETDB1), all LINE (LINE-mm10), SETDB1 overlapping LTR (LTR-SETDB1), and all LTR elements (LTR-mm10)

Next, we analyzed available ChIP-seq datasets from mESC cells for SETDB1^31^, H3K9me3^32^, and H3K14ac^33^ to investigate the in vivo genome binding of SETDB1. As shown in Fig. 6a, we observed co-occurrence of these signals at many sites. Peak calling and analysis of the overlap of peaks and peak regions confirmed a strong enrichment of SETDB1 at H3K9me3 peaks (Fig. 6b), which is in agreement with the literature findings^31,32^. This correlation was expected, because H3K9me3 is the methylation product of SETDB1. However, we also found that 14% of all SETDB1 peaks overlap with H3K14ac peaks and 28% of all H3K14ac peaks overlap with H3K9me3 peaks (Fig. 6b). Strikingly, we observed that 9.7% of all SETDB1 peaks (2231) overlap with H3K9me3 and H3K14ac peaks, which is a strong enrichment, because only 3.9% were expected by chance (=28% of 14%). Further evaluation of the significance of all binary and ternary overlaps was performed by Fisher’s exact test, considering the size and number of regions in the data sets. This resulted in highly significant two-sided *p*-values in all cases (always *p* < 1E-300). In addition, the high significance of the overlap of H3K14ac peaks with the intersection of SETDB1 and H3K9me3 peaks was confirmed by randomization of the H3K14ac regions (Supplementary Note 2).

Clustering of the data on SETDB1 peaks showed that H3K9me3 signals were enriched at or near the vast majority of the SETDB1 binding sites (Fig. 6c, clusters 1–7), and two clusters showed co-occurrence of SETDB1 and H3K9me3 with H3K14ac: cluster 6 (strongly) and cluster 7 (weakly). Cluster 6 contains 12.2% of all SETDB1 peaks, which is in line with the analysis presented above. We conclude that K14ac is present at a subset of SETDB1 binding sites. Prompted by the documented function of SETDB1 to introduce H3K9 methylation at repeats, we analyzed the overlap of the SETDB1 peaks in the different clusters with repeats (Fig. 6d). We observed an enrichment of LTRs in clusters 2–4 (strongly), 7 and 8 (moderately). Moreover, cluster 6, containing regions with SETDB1, H3K9me3, and H3K14ac signals, showed a very strong enrichment of LINE elements. We conclude that a subset of SETDB1 binding sites contains the H3K9me3/H3K14ac double modification and these sites are enriched in LINE elements. Next, we calculated meta-profiles of SETDB1 read density using LTR and LINE elements overlapping with SETDB1 peaks (Fig. 6e). These profiles clearly show that the binding of SETDB1 to LTRs and LINEs differs. While SETDB1 binds to LTRs in the center of the elements, LINEs are bound at the edges. Further analyses showed that H3K9me3 density mirrors SETDB1 density on repeat elements overlapping with SETDB1 peaks (Supplementary Fig. 9a). On LINE elements, a weak H3K14ac signal was detected as well, which (similar to the SETDB1 signal) was centered at the edges of the LINE elements. In agreement with our analysis, the H3K14ac signal was strongly increased on LINE elements from cluster 6 (Supplementary Fig. 9b). The clear H3K14ac enrichment at the flanks of SETDB1 overlapping LINE elements (in particular from cluster 6) strongly supports our conclusion that H3K14ac is involved in the targeting of SETDB1.

### Sub-nuclear localization of 3TD-binding pocket mutants

**Fig. 7:**
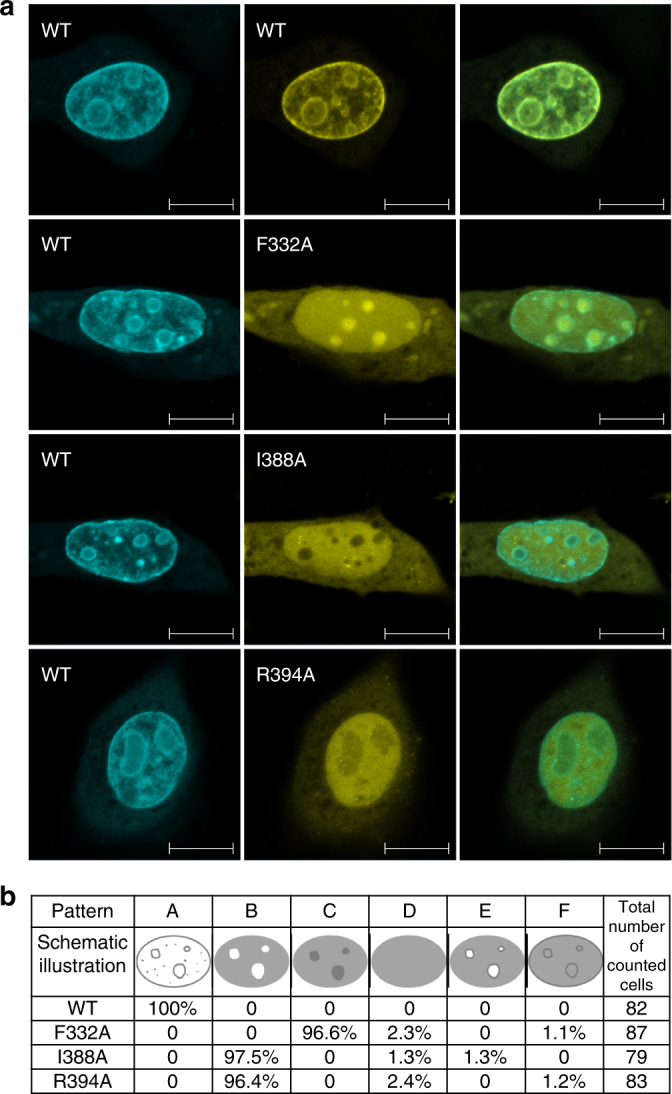
Subnuclear localization of 3TD and 3TD mutants. Pairs of 3TD proteins were co-expressed as fusion constructs with mCerulean (shown in cyan) or mVenus (shown in yellow) in mouse NIH3T3 cells. An overlay of both signals is shown in the third column. **a** Exemplary images of wildtype/wildtype controls (top row) showing a perfect overlay of the two fluorescence signals. In contrast, images of mutant/wildtype combinations clearly show differences in the sub-nuclear localization of the mutants. Scale bar: 10 µm. Additional images are shown in Supplementary Figs. 10 and 11. **b** Summary of the localization data. Around 80 cells were inspected per experiment, classified to different phenotypes and counted. The following phenotypes were used: A (wildtype like)-granular, perinucleolar with nucleolar exclusion, B-diffused with nucleolar exclusion, C-diffused with nucleolar enrichment, D-diffused, E-diffused, perinucleolar with nucleolar exclusion, F-diffused, perinucleolar with nucleolar enrichment. Phenotypes D–F were observed very rarely

Finally, we investigated if the sub-nuclear localization of 3TD in mouse NIH3T3 cells depends on its H3 tail interaction (Fig. 7 and Supplementary Figs. 10 and 11). 3TD and 3TD mutants were expressed as fusion constructs with mCerulean or mVenus in mouse NIH3T3 cells. Wildtype 3TD showed a granular distribution with perinucleolar enrichment. As expected, co-expression of two wildtype constructs resulted in a perfect overlay of the two fluorescence signals. In contrast, all mutants showed clear differences in their sub-nuclear localization. F332A displayed a diffused localization with enrichment in nucleoli, while I388A and R394A showed diffused localization with nucleolar exclusion. These data show that substitutions of single residues important for peptide binding (F332, I388, or R394) changed the sub-nuclear localization of the mutants, indicating that H3 tail binding affects the sub-nuclear localization of 3TD. We also investigated the contribution of 3TD to the localization of the full-length SETDB1, but quick and efficient export of SETDB1 from the nucleus, consistent with previous reports^34,35^, prevented us from drawing any conclusions.

## Discussion

It is well established that epigenetic writers frequently also contain reading functions^36^. Often the modification introduced by the writer is read by the reader, as exemplified in the cases of the SUV39H1^37^ and G9a H3K9 PKMTs^38^, a process that can contribute to propagation and spreading of the mark and by doing so to ensure stability of the epigenome network. However, under specific conditions, efficient switching mechanisms are required to change functional chromatin states. Under these circumstances, recruitment of the required writers would depend on the reading of an “opposing” signal, as discovered in this work for the SETDB1 H3K9 trimethylase. We show here that the 3TD domain of SETDB1 specifically binds to histone H3 tails containing a combinatorial modification at K9 and K14. The activating modification, H3K14ac, is recognized as a primary mark responsible for binding, and must be accompanied by methylation of H3K9, which is associated with active or repressed chromatin depending on the methylation state. Co-occurrence of K9 methylation and K14 acetylation had been shown previously by mass spectrometry in histone H3 isolated from different human cell types (Supplementary Fig. 12)^39,40^, but the functions of these double marks are not known. We show activity of SETDB1 on H3K14-acetylated substrates in peptide array methylation experiments, suggesting that recruitment of SETDB1 to H3K9me/K14ac chromatin can result in silencing of the target regions, by introduction of H3K9me3 via the SET domain of SETDB1 and by deacetylation of K14ac via SETDB1-associated HDAC activity. This finding is in agreement with the observation that H3K9me3 levels decrease on H3 tails with acetylated K14 after the combined knock-down of the three H3K9 methyltransferases EHMT1, EHMT2, and SETDB1^40^, and suggests that SETDB1 is responsible for this effect.

**Fig. 8:**
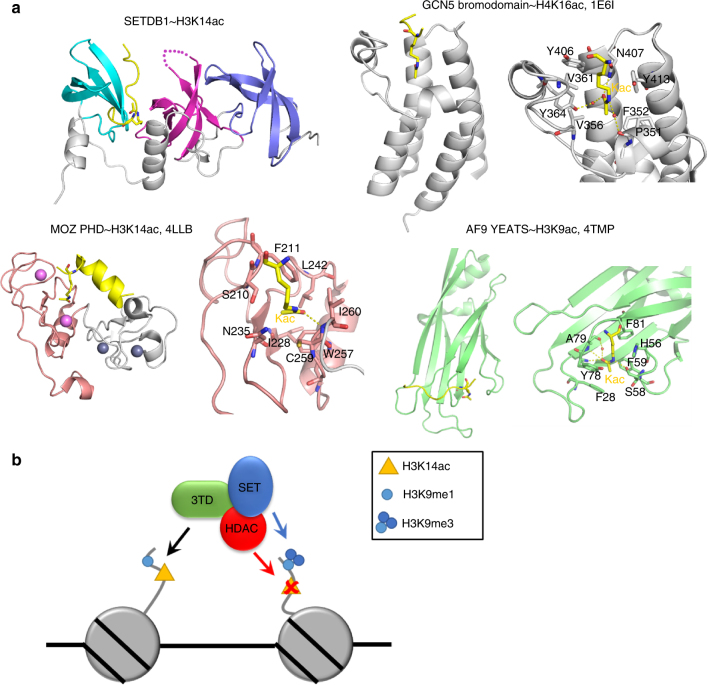
Overview of Kac binding domains and model of the function of SETDB1. **a** Comparison of the 3TD Kac binding site with Kac binding to the GCN5 bromodomain, MOZ PHD domain, and AF9 YEATS domain. **b** Model illustrating the putative role of 3TD in the function of SETDB1. Binding of 3TD to H3 tails containing K9me–K14ac recruits the SETDB1 complex to these regions. Afterward, the SET domain of SETDB1 introduces H3K9me3, and depending on the complex composition, the HDAC activity of NuRD may remove H3K14 acetylation, finally leading to the silencing of the genomic region

The three Tudor modules of SETDB1 fold as a tightly connected protein domain. Binding of K14ac occurs at the interface between TD2 and TD3 and is mediated by hydrogen bonding and hydrophobic interactions. Previously, the bromodomain has been characterized as an acetyl-lysine binding module^41^, and tandem PHD fingers of DPF3b^42^ and MOZ^43–45^ have also been reported to recognize H3K14ac. The MOZ tandem PHD finger in addition interacts with acylated lysine^46^. Moreover, the YEATS domains of AF9 and Taf14 were reported to recognize H3K9ac^47,48^ as well as lysine crotonylation^49,50^. In contrast, Tudor domains have been identified as methyl-lysine or methyl-arginine reading domains^26,27^. Our biochemical and structural analyses demonstrate that 3TD of SETDB1 functions as a recognition domain for acetyl-lysine (Fig. 8). Previously, binding to a similar bivalent H3K9me3/H3K18ac modification has been observed for TRIM33^51^. However, in that case the binding is mediated by two separate reading domains (one PHD domain and one bromodomain), which need to reside in a greater distance to one another to allow for specific binding of H3K9me3/H3K18ac to happen. The observation that TRIM33 binding to H3K9me3/H3K18ac activates gene expression, while SETDB1 binding to H3K9me/K14ac likely leads to gene silencing (because H3K9me3 is introduced and K14ac could be removed), suggests that these combinatorial K9me/Kac modification states have distinct roles. Interestingly, the conformation of the peptide bound to 3TD depends on the modification state of K9. While K9me3 binding occurs at the TD2 domain with higher affinity, TD3 preferentially recognizes K9me1 and K9me2 substrates—illustrating a pocket switch mechanism in a histone reading domain. It is conceivable that this conformational change of the bound peptide triggers downstream effects in SETDB1, for example allosterically modulating its activity or controlling the interaction with its complex partners, like KAP-1^9^, the NuRD complex^19–21^, DNMT3A^17^, other H3K9 PKMTs^18^, or the HUSH complex^16^. Future work will be needed to explore these effects in more detail.

Our data suggest that H3K9me and K14ac define a bivalent chromatin state, which is bound by 3TD of SETDB1, in agreement with other examples documenting roles of SETDB1 at bivalent chromatin^32,52^. By this process, SETDB1-mediated silencing of endogenous retroviral elements might be connected to the activating H3K14ac modification, when it co-occurs with methylated H3K9. After recruitment of SETDB1 to H3K9me1/K14ac or H3K9me2/K14ac sites, SETDB1 is expected to methylate them to the K9me3 state (Fig. 8b), which is in agreement with the binding of SETDB1 at LINE elements containing K9me3/K14ac. Depending on the exact composition of the SETDB1 complex bound, deacetylation of K14ac could also be catalyzed, suggesting that SETDB1 bound to K9me3 marked LTRs initially could have been recruited by K14ac, but this modification has already been removed. Recruitment by K14ac might help to focus SETDB1 activity on the most active LTR and LINE elements, supporting the silencing of active and hence most dangerous repeat elements. In agreement with this model, the SETDB1-mediated silencing of endogenous retroviral elements has been also linked to histone variant H3.3, which is another signal often associated with gene activation^53^. Hence, the SETDB1 silencing system might be specifically attracted to repeat elements decorated with activating marks, such as H3.3 and H3K14ac. In summary, our data suggest that recruitment of SETDB1 to H3K9me/H3K14ac might allow to efficiently counteract acetylation of these elements, introduce K9 trimethylation, and ensure silencing of retrotransposons containing internal activating elements. Future work may include the generation of endogenous SETDB1 mutants containing amino acid exchanges in 3TD by CRISPR/Cas9-mediated homologous recombination and subsequent studies of the biological consequences of these exchanges.

## Methods

### Protein expression and purification

Wildtype SETDB1–3TD was cloned into the pET28a-MHL (residues 190–410), pGEX6P2, and pGEX-4T-1 (residues 197–403) vectors. Point mutants were generated by site-directed mutagenesis. Wildtype and mutant proteins were expressed in an SGC-generated derivative strain of BL21 *Escherichia coli* with the pRARE plasmid for overcoming the codon bias. Cells were grown in TB medium or in M9 minimal medium supplemented with ^15^NH_4_Cl for the NMR analyses. Expression was induced with 0.5 mM IPTG, and the bacteria were harvested by centrifugation and lysed by sonication. The GST-fusion proteins were purified using Glutathione-Sepharose 4B beads (GE Healthcare). The GST-tag was either cleaved with PreScission protease, or left for the purposes of western blot analysis or peptide array probing, in which case the GST-fusion protein was eluted off the Glutathione-Sepharose beads using 50 mM reduced L-glutathione (Sigma-Aldrich). The 6×His-tagged protein was purified by an Ni-NTA column (Qiagen) followed by a gel filtration column (Superdex 75, GE Healthcare). For SETDB1 methylation kinetics, an N-terminus-deleted construct was cloned into the pFBOH-SUMOstar-TEV vector as 6×His-SUMO-TEV-SETDB1 (190–1291) fusion protein. The resulting virus was transformed into Sf9 insect cell for protein expression, and purified using Ni-NTA column (Qiagen) followed by ion exchange chromatography (Hitrap Q HP column).

### CD spectroscopy

Folding of purified proteins was analyzed by circular dichroism (CD) spectroscopy. CD measurements were performed using a J-815 circular dichroism spectrophotometer (JASCO Corporation, Tokyo, Japan) using GST-tagged 3TD domains in 100 mM KCl, 5 mM HEPES pH 7.5, and 5% glycerol. Depending on the protein preparation, spectra were measured at 10 µM or 2 µM protein. The spectra were collected at 22 °C using a 0.1-mm cuvette in a wavelength range between 195 and 240 nm. For each sample, at least 60 scans were collected and averaged.

### Protein crystallization

Purified proteins mixed with a 3- to 5-fold stoichiometric excess of the respective peptides were crystallized using the sitting drop vapor diffusion method at 18 °C by mixing 0.5 µl of the protein with 0.5 µl of the reservoir solution. The 3TD bound with H3K9me2/K14ac or H3K9me3/K14ac complex was crystallized in 25% PEG 3350, 0.2 M NaCl, 0.1 M Hepes pH 7.5, and 5% glycerol. The 3TD–H3K9me0/K14ac complex was crystallized in 20% PEG 5000 monomethyl ether, 0.1 M Bis-Tris pH 6.5. The 3TD W358A mutant bound with H3K9me2/K14ac or H3K9me3/K14ac complex was crystallized in 25% PEG 3350, 0.2 M Li_2_SO_4_, and 0.1 M Hepes pH 7.5. Further details of crystallization and structure determination are given in Supplementary Note 1.

### Nuclear magnetic resonance spectroscopy

NMR experiments were collected on a Bruker Avance 500-MHz spectrometer equipped with a cryogenic probe. Chemical shift perturbation experiments were carried out using uniformly ^15^N-labeled wildtype 3TD protein in 20 mM Tris/HCl pH 7.0 and 150 mM NaCl. ^1^H, ^15^N HSQC spectra were recorded at 25 °C in the presence of increasing concentrations of histone tail peptides. *K*_d_ values were calculated by a nonlinear least-squares analysis in Kaleidagraph using the equation

$$\Delta \delta = \Delta \delta _{\max }\left( {\left( {c_{\rm L} + c_{\rm P} + K_{\rm d}} \right) - \sqrt {\left( {c_{\rm L} + c_{\rm P} + K_{\rm d}} \right)^2 - 4c_{\rm L}c_{\rm P}} } \right)/2c_{\rm P},$$

where *c*_L_ is the concentration of the peptide, *c*_P_ is the concentration of the protein, Δ*δ* is the observed normalized chemical shift change, and Δ*δ*_max_ is the normalized chemical shift change at saturation, calculated as

$$\Delta \delta = \sqrt {\left( {\Delta \delta _{\rm H}} \right)^2 + \left( {\Delta \delta _{\rm N}/5} \right)^2},$$

where *δ* is the chemical shift in p.p.m.

### Isothermal titration calorimetry

ITC experiments were carried out at 25 °C on a VP-ITC calorimeter (MicroCal). Protein and peptide were kept in an identical buffer of 20 mM Tris/HCl pH 7.0 and 150 mM NaCl. ITC measurements were carried out with protein and ligand concentrations ranging from 50 to 100 μM and 1–2 mM, respectively. Binding isotherms were analyzed by nonlinear least-squares fitting of the data using Microcal ORIGIN software (Microcal) using a one-site binding model.

### Mass spectrometry

3TD pull-down and input control mononucleosomal chromatin isolated from HEK293 cells were resolved on 15% SDS-PAGE gels. The bands corresponding to histone H3 were excised from the gels and shredded into small 1-mm cubes. Coomassie, detergents, and other chemicals that could interfere with trypsin digestion or MALDI analysis were removed in three subsequent 15-min incubation steps with 100 µl of 1:1 (v/v) mixture of 50 mM ammonium bicarbonate and acetonitrile. After each incubation, the gel pieces were dehydrated by addition of 100 µl pure acetonitrile and the liquid was discarded after each wash. For in-gel digestion, the gel pieces were rehydrated in 50 µl 25 mM ammonium bicarbonate containing 5 ng/µl sequencing-grade modified trypsin (Promega) and incubated at 37 °C for 30 min. Afterward, the supernatant containing the digested peptides was transferred into a new tube, flash frozen in liquid nitrogen for later use, or directly spotted on an AnchorChip (Bruker) target plate, dried, and covered with alpha-cyano-4-hydroxycinnamic acid (HCCA). Peptide mass fingerprinting was done on an AutoFlex Speed MALDI TOF/TOF mass spectrometer (Bruker) operated in reflected positive mode. The spectra were recorded between 500 and 4000* m*/*z*. Peptide calibration standard (Bruker) was used for mass calibration. The obtained spectra were analyzed with the BioTools package (Bruker). For MS/MS analysis, selected peaks were fragmented and measured in LIFT mode, the mass spectra were matched against possible peptides found in nonredundant (nr) database using MASCOT software, or matched to the theoretical sequence of the peptide identified in the first PMF analysis.

### Cell lines

Human HEK293 and mouse NIH3T3 cells were obtained from the Leibniz Institute DSMZ-German Collection of Microorganisms and Cell Cultures. The mouse ES cell line E14 (129/Ola) was obtained from Wolf Reik (Cambridge, UK). All cells were free of mycoplasma.

### Binding and methylation of peptide arrays

Experiments to determine the binding specificity of 3TD wildtype and mutants were performed using CelluSpots peptide arrays (MODified Histone Peptide Array, Active Motif), as described previously, in 20 mM HEPES pH 7.5, 100 mM KCl, 1 mM EDTA, 0.2 mM DTT, and 10% glycerol^54^. All peptide array binding studies were conducted at least in two technical repeats. Annotations of all spots are given in Supplementary Table 2. The amounts of GST-fused Tudor proteins used in peptide array binding and western blot analyses ranged from 0.5 to 100 nM. For methylation analysis, MODified Histone Peptide arrays (Active Motif) were incubated with 20 nM SETDB1 in buffer (50 mM Tris/HCl pH 9.0, 4 mM DTT, 5 mM MgCl_2_) supplemented with 0.38 µM radioactively labeled [methyl-3H]-AdoMet (Perkin Elmer) for 2 h at room temperature. The arrays were then washed 5 times with wash buffer (100 mM NH_4_HCO_3_ and 1% SDS), incubated in Amplify NAMP100 (GE Healthcare) for 5 min, and then exposed to Hyperfilm TM ECL high-performance chemiluminescence film (GE Healthcare) in the dark at −80 °C for 1–7 days.

### Binding to histones and histone tails

Native histones were isolated by acid extraction^55^ from HEK293 cells, and recombinant histone H3 was purchased from New England Biolabs. For the experiment with HDAC inhibitors, cells were treated for 48 h with 0.4 mM TSA or 5 mM sodium butyrate. Five micrograms of native histones and matching amount of recombinant H3 were electrophoresed on 18% SDS-PAGE and transferred onto nitrocellulose membranes. The membrane was then blocked in 5% milk in TBST and incubated with purified GST-tagged 3TD, followed by the anti-GST (GE Healtcare 27457701A, dilution 1:5000) and anti-goat antibody (Sigma A4174, dilution 1:10000). The amount of GST-fused Tudor protein used in western blot analyses was 100 nM. All experiments were performed in duplicates or triplicates. In the western blots, following antibodies were used: anti-K14ac (Millipore MABE351, dilution for checking specificity: 1:2000 or 1:1000; for western blot: 1:500), anti-H3K9me3 (Abcam ab8898, dilution 1:2000), anti-H3K9me2 (Abcam ab1220, dilution 1:1000), and anti-H3K4me3 (Abcam ab8580, dilution 1:2000). For specificity analysis of antibodies, see Supplementary Fig. 13. Secondary antibodies were anti-rabbit IgG HRP (GE Healtcare NA934, dilution 1:5000) and anti-mouse IgG HRP (GE Healtcare NXA931, dilution 1:5000). Native histone and nucleosome pull-down experiments were done at least in two biological repeats. All antibodies were used following the manufacturer’s recommendations. Uncropped gel images are shown in Supplementary Fig. 14.

### Analysis of SETDB1 genomic binding

SETDB1, H3K9me3, and K14ac ChIP data from murine embryonic stem cells were from GSE17642^31^, GSE18371^32^, and GSE31284^33^, respectively. The raw reads were mapped to mm10 with Bowtie2, using default settings^56^ and RPKM normalized. Peaks were called with MACS^57^ in the Galaxy environment^58^. The quantitative analyses of peak overlap were carried out in Galaxy using the Intersect tools. ComputeMatrix and PlotHeatmap in DeepTools was used for generation of heatmaps, meta-profiles, and K-means clustering^59^. Statistics were determined using the Fisherʼs Test and ShuffleBED tools in DeepTools. Overlap regions were checked not to include blacklisted regions for mm10 downloaded from ENCODE. LINE and LTR elements were downloaded from NCBI table browser. The Integrative Genomics Viewer was used for visualization of the data and preparation of browser shots^60^. For annotation of repeats and known chromatin modifications to genomic regions, EpiExplorer was used^61^.

### Fluorescence microscopy

For sub-nuclear localization studies, SETDB1 3TD wildtype and mutant domains were cloned in expression constructs containing an NLS and fused to mCerulean and mVenus. NIH3T3 cells were seeded on tissue culture dishes with glass bottom and 1 day later co-transfected with plasmids expressing one mCerulean and one mVenus tagged 3TD wildtype or mutant domain using FuGENE HD (Promega) according to the manufacturerʼs instructions. After 24 h, images were collected using a Zeiss LSM 710 confocal microscope and Plan-Apochromat 63×/1.40 Oil DIC M27 objective.

### Data availability

Structural data have been submitted to the PDB database under accession numbers 6BHD, 6BHE, 6BHG, 6BHH, and 6BHI. All other data supporting the findings of this study are available within the paper and its supplementary information files. Additional primary data are available from the corresponding authors upon reasonable request.

## Electronic supplementary material

Supplementary Information
