# Supplementary material for: H3K14ac is linked to methylation of H3K9 by the triple Tudor domain of SETDB1
Source: Nat Commun. 2017 Dec 12;8:2057. doi: 10.1038/s41467-017-02259-9 (PMC5727127; doi:10.1038/s41467-017-02259-9)
Supplement: Supplementary file 1 — Supplementary Information [file 41467_2017_2259_MOESM1_ESM.pdf]

## Supplementary figures

**Supplementary Figure 1: Peptide binding of 3TD.** (**a**) Titration of 3TD binding to peptide arrays. Note the specific binding to H3K9me1/2/3-K14ac double modified peptides. Binding to monomodified H3K14ac was observed only at the highest 3TD concentration (100 nM). (**b**) ITC analysis of 3TD binding to single modified H3 tail peptides. Data are summarized in Fig. 2b. (**c**) NMR chemical shift perturbation experiments carried out to detect potentially weak binding of 3TD to H3K14ac, H3K9me3 and unmodified H3. Weak binding of H3K14ac could be detected and the binding constant determined, but no detectable binding of H3K9me3 and unmodified H3 was observed. Panel **a** relates to Fig. 1, panels **b** and **c** to Fig. 2.

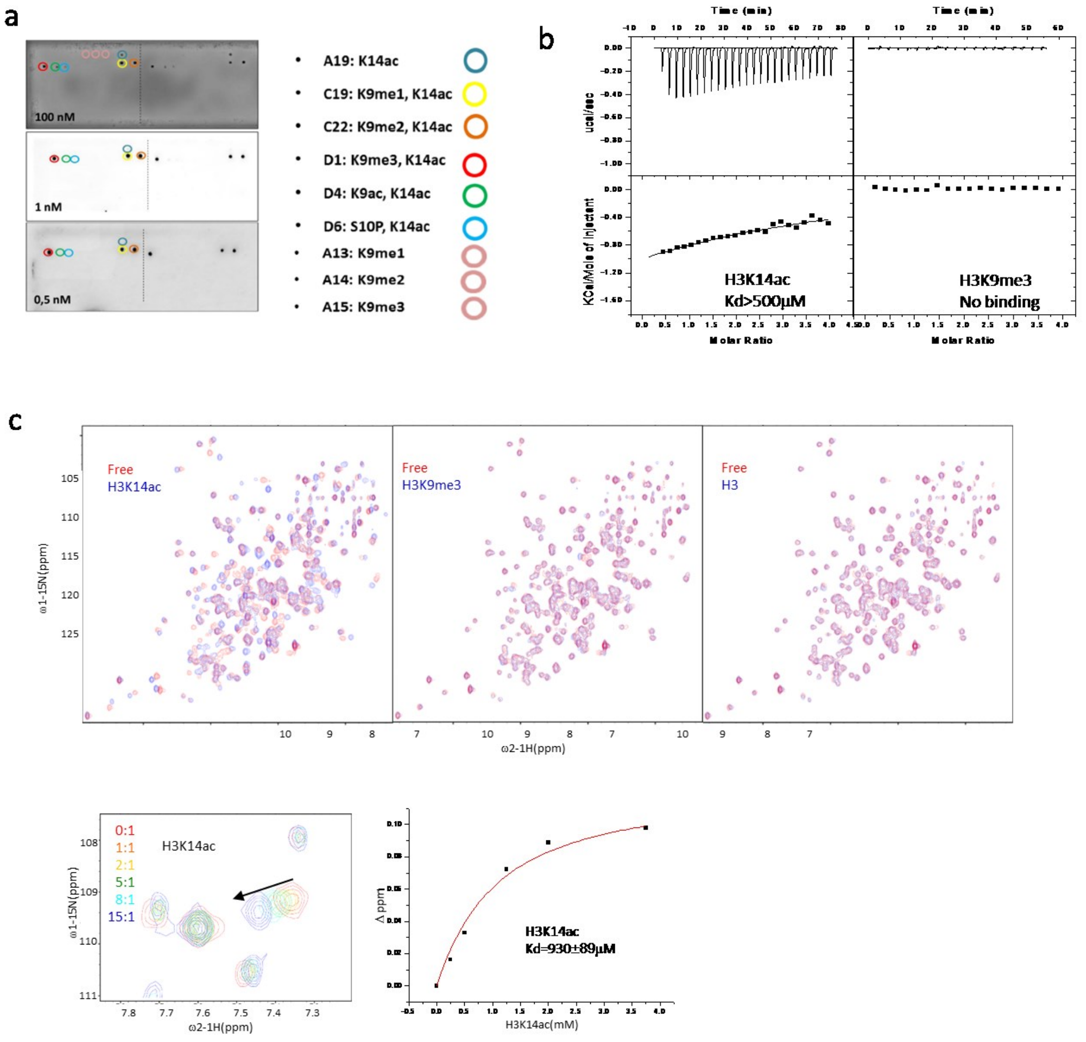

**Supplementary Figure 2: Structural analysis of 3TD.** (**a**) Overall arrangement of the individual Tudor domains in 3TD (apostructure: 3DLM). (**b**) Sequence alignment of 3TD regions in different species. Stars indicate residues interacting with K14ac, triangles indicate residues forming the aromatic cage in TD2 and TD3. (**c**) View of the incomplete aromatic pocket in TD1. This figure relates to Figs. 1a, 3 and 4.

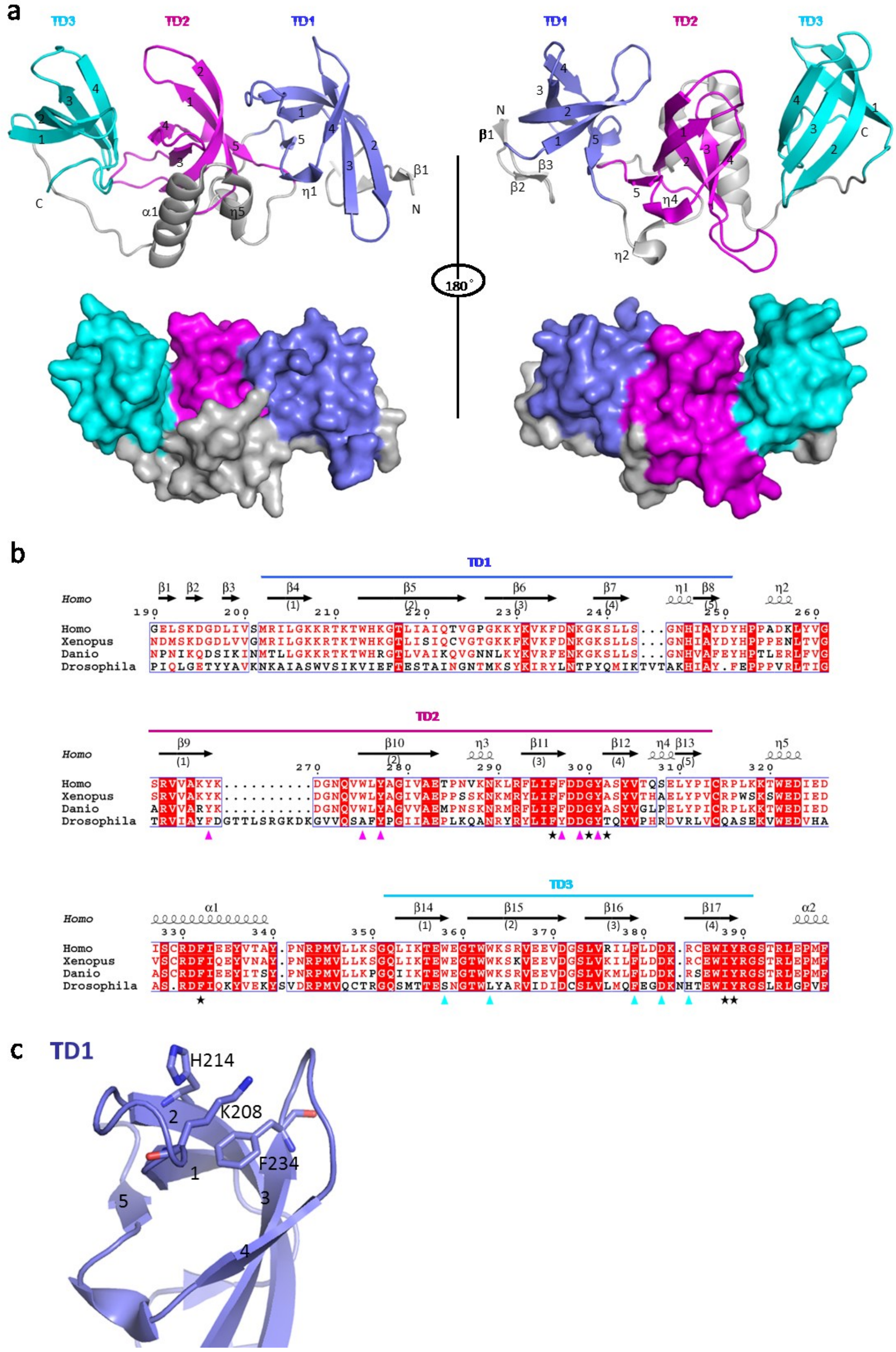

**Supplementary Figure 3: Details of the structures of 3TD and W358A interacting with histone peptides.** mFo-DFc omit maps for the peptide ligands were calculated with PHENIX and are displayed as a green mesh at level "3". This figure relates to Fig. 3 and 4.

3TD-H3K9me0K14ac

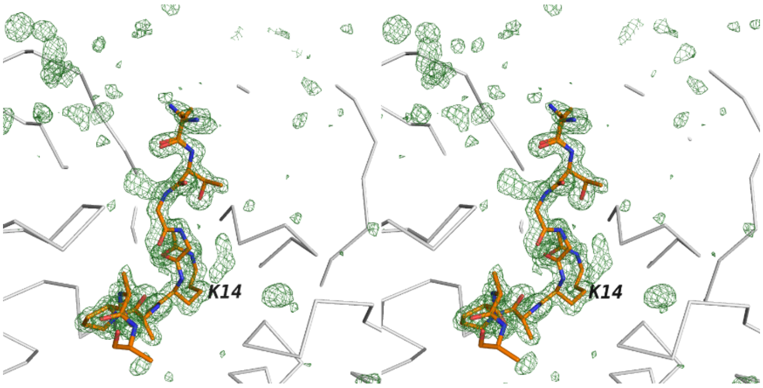


3TD-H3K9me2K14ac

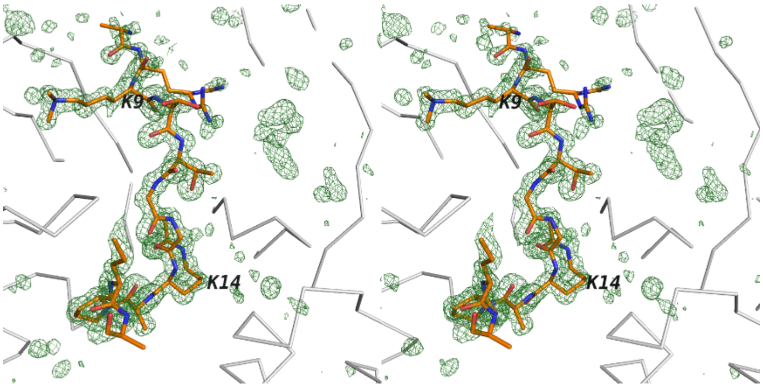


3TD-H3K9me3K14ac

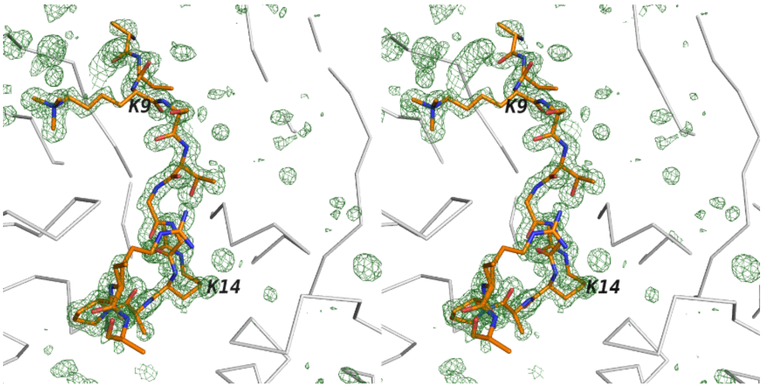

3TD(W358A)-
H3K9me2K14ac

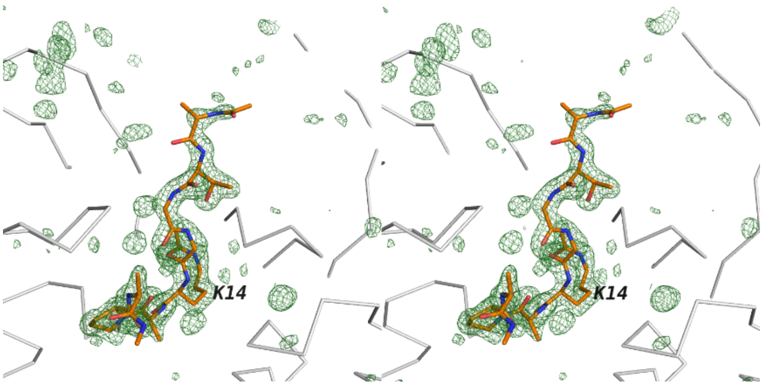


3TD(W358A)-
H3K9me3K14ac

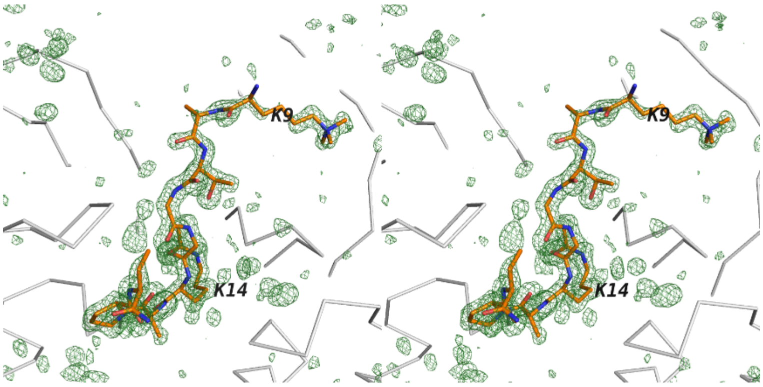

**Supplementary Figure 4: Modified histone peptide array binding experiments with 3TD and 3TD mutants.** (**a**) Mutations at the acetyl binding pocket. (**b**) Mutations in the TD1 domain. (**c**) Mutations at the methyllysine binding pockets in TD2. (**d**) Mutations at the methyllysine binding pockets in TD3. (**e**) Summary of the results of mutations in TD1, TD2 and TD3 illustrating the complementary effects of mutations in TD2 and TD3 on the specificity of binding to K9 methylated peptides. Data for Y268A and W358A are shown in Fig. 1 and were included for comparison. Correct folding of purified proteins was verified by CD spectroscopy (Supplementary Fig. 5). This figure relates to Fig. 1. Full annotations of all spots in the peptide arrays are given in Supplementary Table 2.

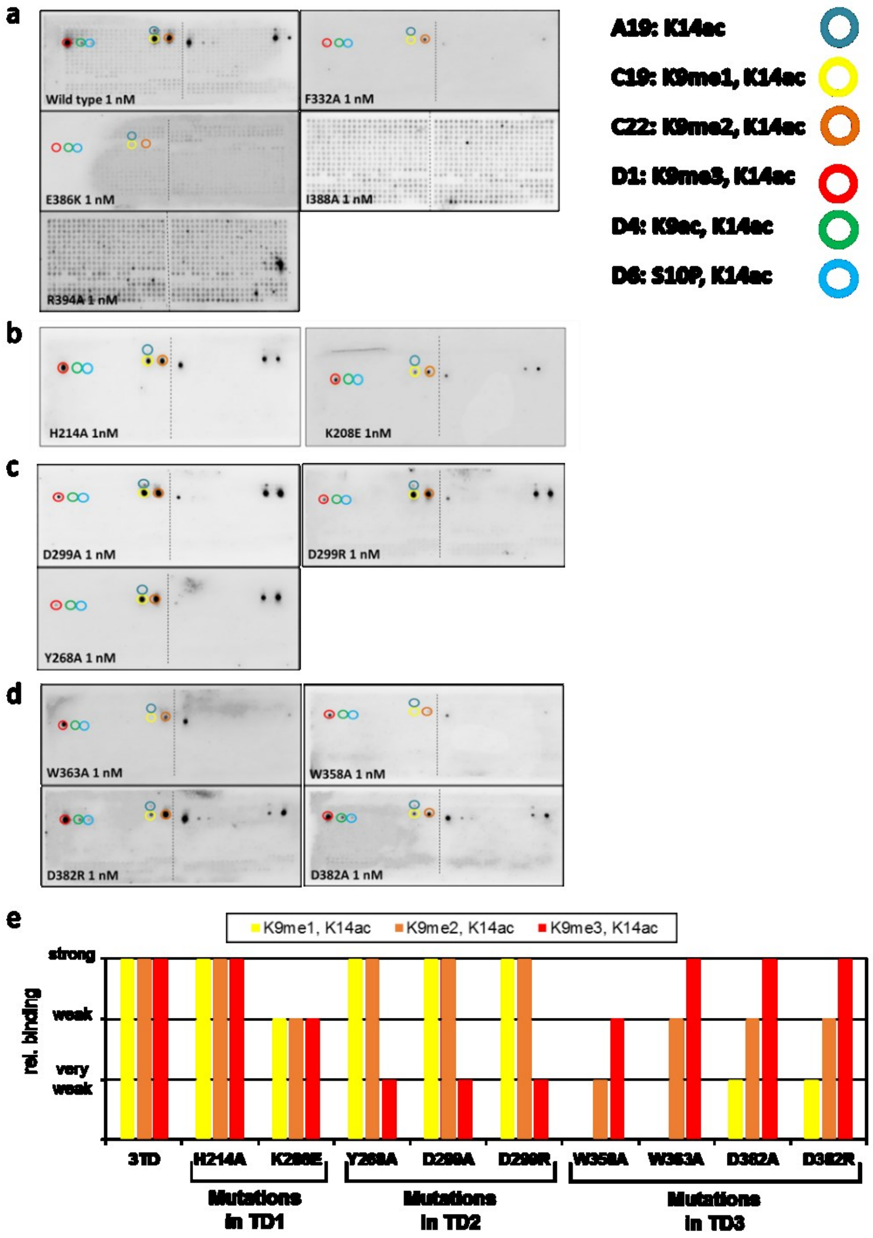

**Supplementary Figure 5: Circular dichroism spectra of 3TD and representative 3TD mutants.** Proteins were purified as GST fusions and the CD spectra determined at protein concentrations of 10 µM (panel **a**) and 2 µM (panel **b**). All CD spectra were superimposable within the level of noise indicating a similar folding of wildtype 3TD and all mutant proteins. This figure relates to Figs. 1 and 2 and Supplementary Figs. 4 and 6.

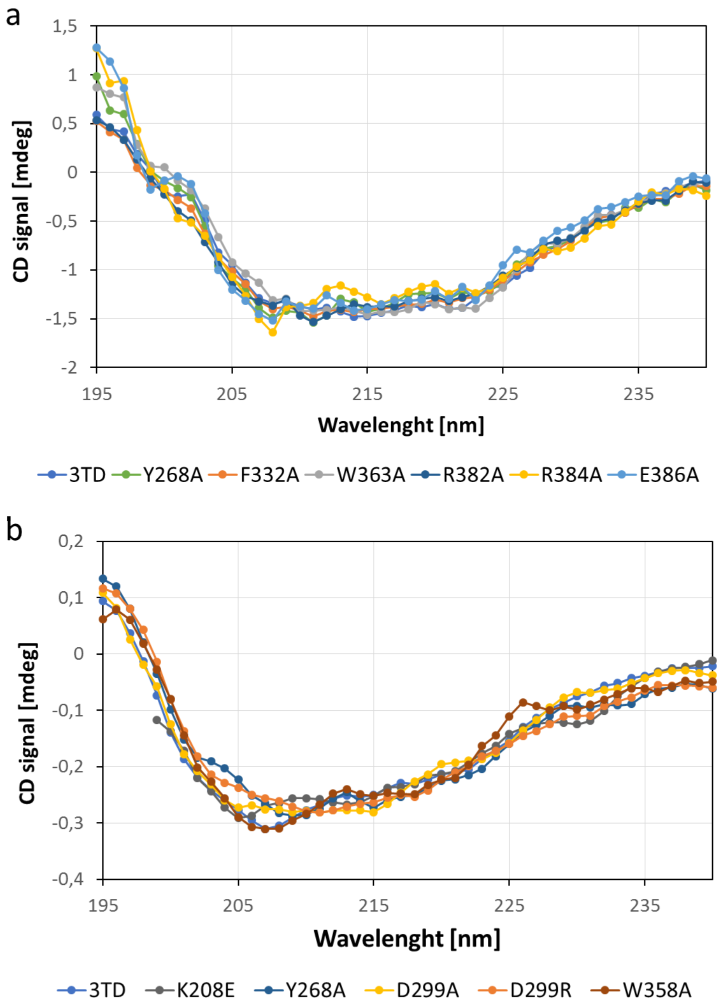

**Supplementary Figure 6: ITC peptide binding experiment with 3TD mutants.** Data are summarized in Fig. 2b. This figure relates to Fig. 2.

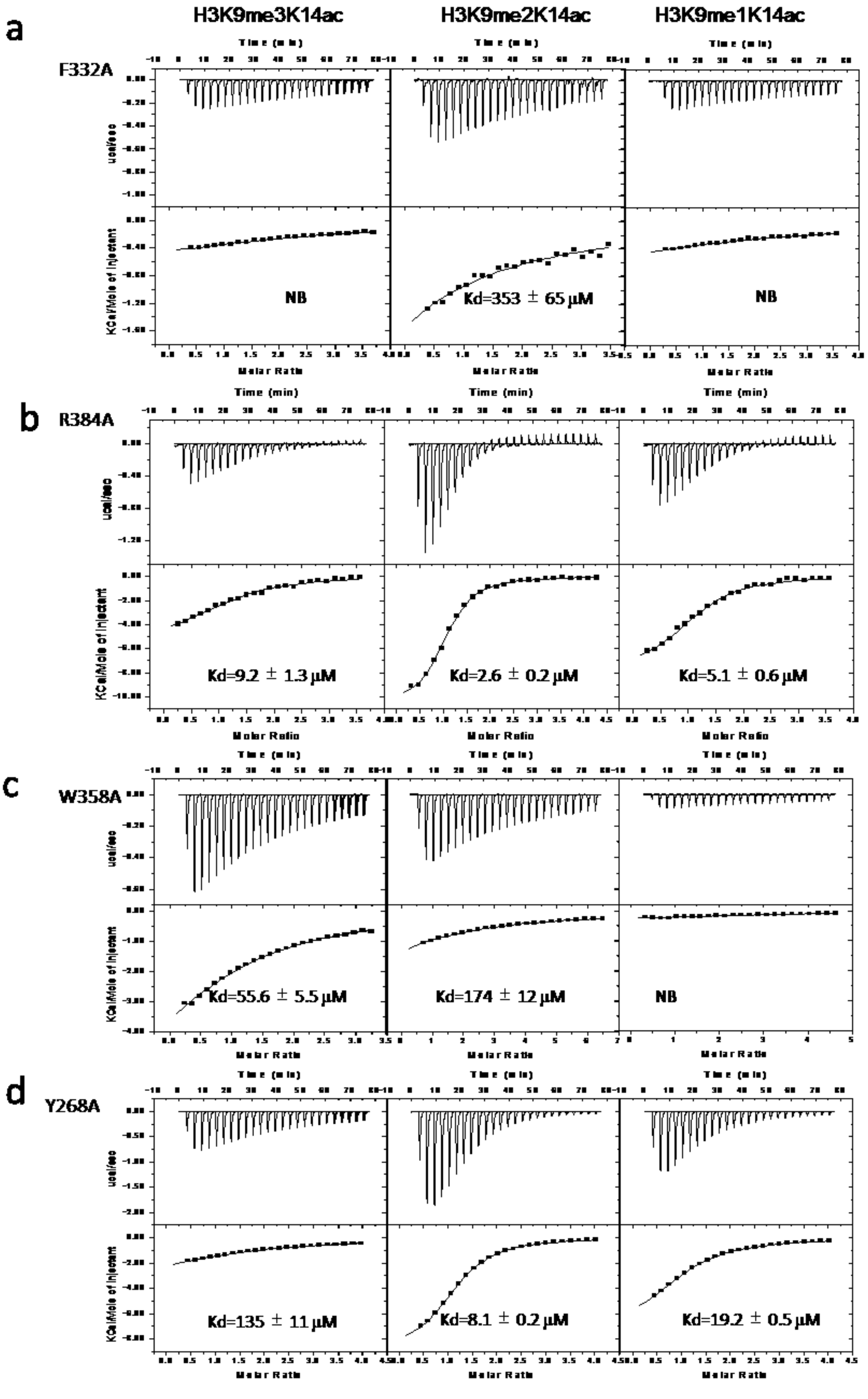

**Supplementary Figure 7: Methylation of modified histone peptide arrays by recombinant SETDB1.** The arrays containing covalently bound histone peptides in various modified forms were incubated with SETDB1 (190-1291) purified from Sf9 cells in the presence of radioactively labeled AdoMet and the transfer of radioactivity to the peptide spots was detected by autoradiography. The arrays contain two copies of 384 individual Histone tail peptides in different methylation states, including 219 H3 1-19 peptides, methylation of which was analyzed here. (**a**) Example image of the methylated arrays. (**b**) Annotation of the spots carrying K9 unmodified, K9me1, K9me2, K9me3, and K9ac peptides. (**c**) Annotation of the spots carrying S10ph, T11ph and S10ph/T11ph peptides, showing that S10ph and T11ph inhibit SETDB1. (**d**) Quantitative analysis of the methylation of K9me0, K9me1 and K9me2 spots with or without K14ac based on 4 experiments (two biological repeats each with two technical repeats, error bars indicate the SD). Peptides containing H3K9me3 and H3K9ac are not methylated. Acetylation of K4 (spot A9) did not influence K9 methylation. The array does not contain a peptide carrying K4 and K14 acetylation. The full annotations of all spots are given in Supplementary Table 2. This figure relates to Fig. 8b.

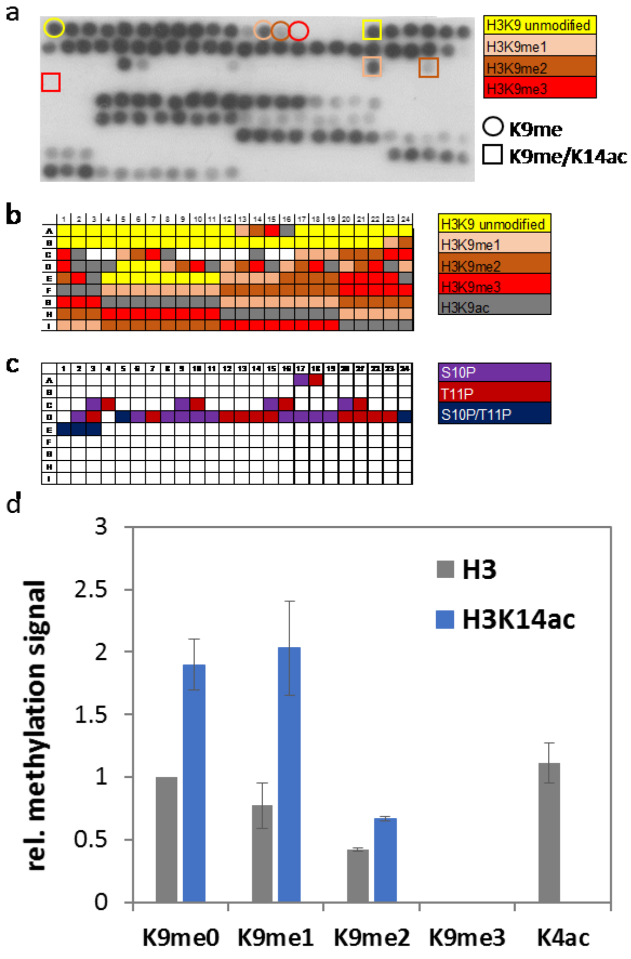

**Supplementary Figure 8: MALDI MS analysis of the 3TD mononucleosomal pull-down.** (**a**) Example of mass spectra shown in the range of 895-1035 Da. The peaks of the H3 (9-17) K9me1/2/3-K14ac peptides are annotated as 1, 2, and 3 in the pulldown image, the unrelated, unmodified H3 peptides used for internal normalization of input and pulldown are highlighted by asterisks. The analysis shows a strong enrichment of the K9me1/2/3-K14ac peptides in the 3TD pulldown as compared to the input. (**b**) Same as in panel **a**, but shown at enhanced x- and y-axis scale. (**c**) MS/MS analysis confirming the identity of the 901.521 Da peptide to be unmodified H3 (9-17). Fragment ions of the a, b, and y series are indicated in green, red and blue. (**d**) MS/MS analysis confirming the identity of the 1032.595 Da peptide to be unmodified H3 (41-49). (**e**) Compilation of all peptides identified in this study. The MS/MS analysis confirming the identity of the H3 (9-17) K9me2-K14ac peptide is shown in Fig. 5e. Peaks corresponding to the monomethylated (915.537 Da), dimethylated (929.552 Da) and trimethylated or acetylated (943.568/943.532 Da) peptides were not detected, neither in input nor pulldown. This figure relates to Fig. 5d and 5e.

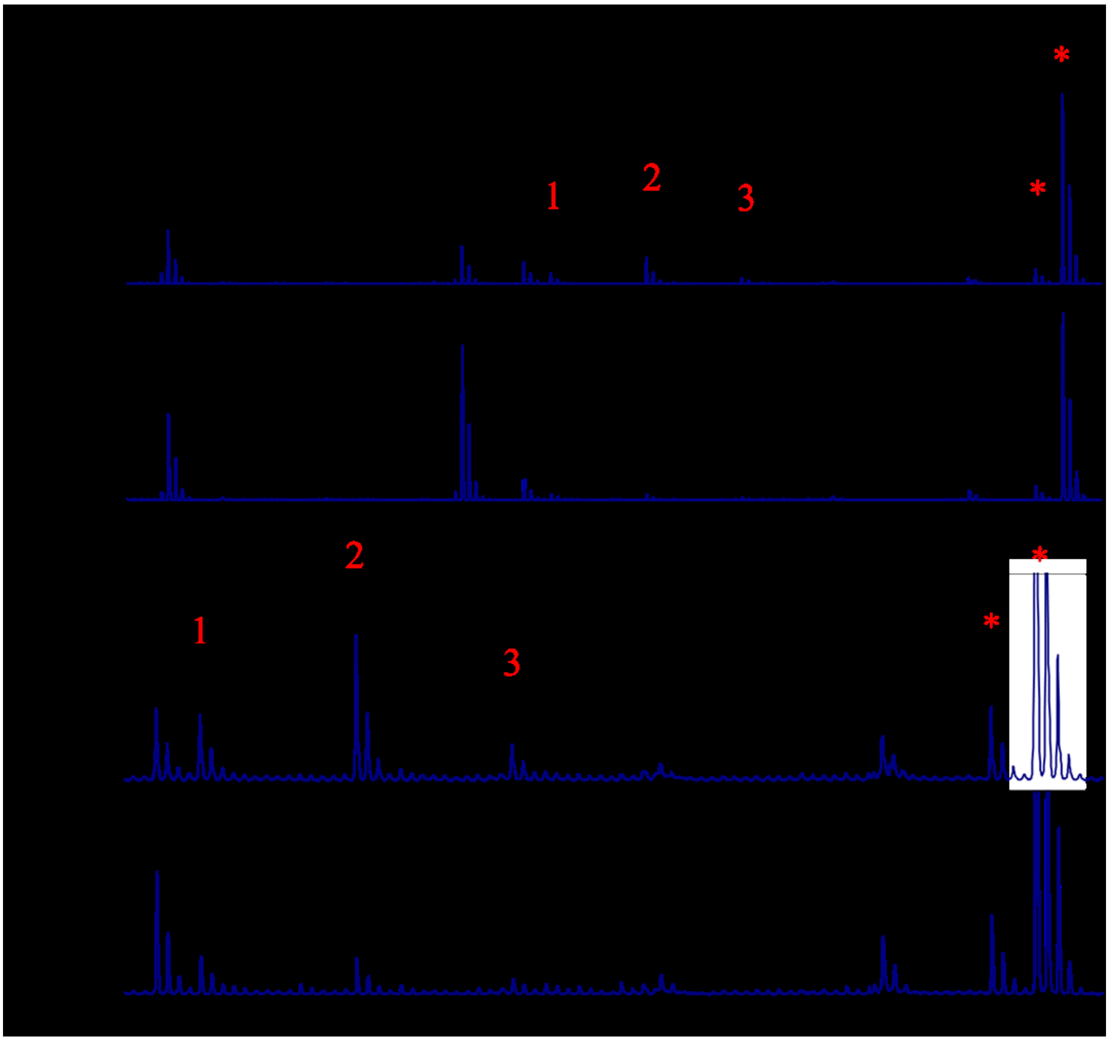

c

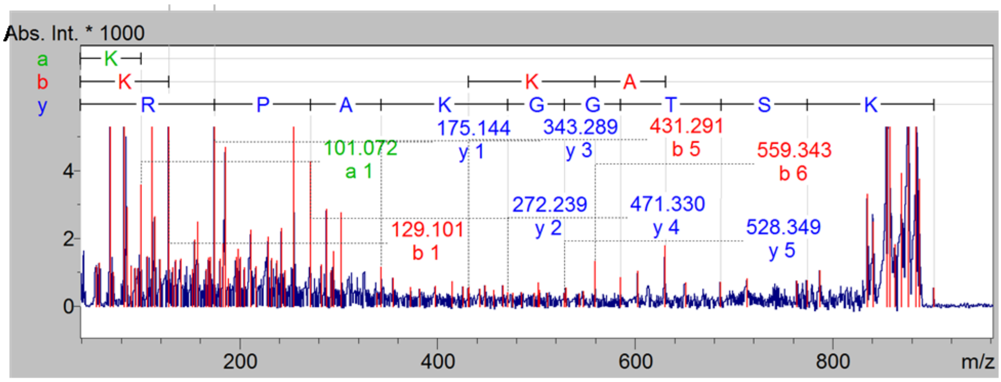


d

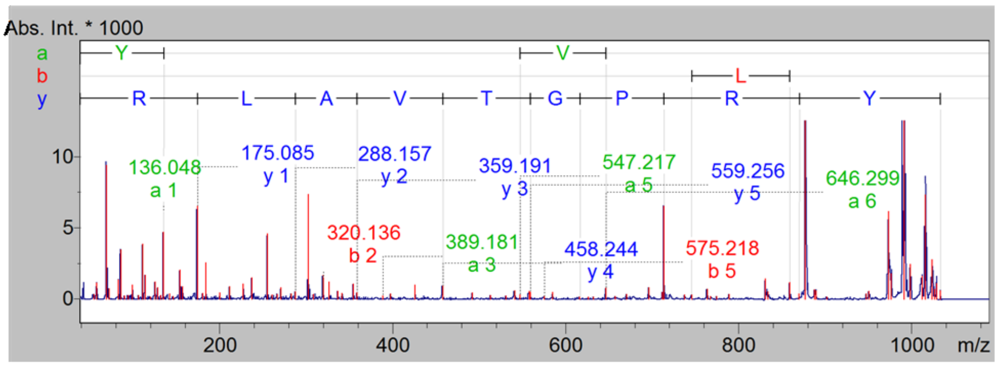


e

| *Obs. mass [Da]* | *Pepide* | *Sequence* | *Theo. mass (MH⁺) [Da]* |
|---|---|---|---|
| 901.509 | H3(9-17) | KSTGGKAPR | 901.521 |
| 957.548 | H3(9-17)K9me1/K14ac | KSTGGKAPR | 957.548 |
| 971.565 | H3(9-17)K9me2/K14ac | KSTGGKAPR | 971.563 |
| 985.573 | H3(9-17)K9me3/K14ac | KSTGGKAPR | 958.579 |
| 1028.612 | H3(65-72) | LPFQRLVR | 1028.636 |
| 1032.592 | H3(41-49) | YRPGTVALR | 1032.595 |

**Supplementary Figure 9: Additional meta-profiles of SETDB1, H3K9me3, H3K14ac and control read density over LINE and LTR repeats**. (**a**) Meta-profiles of SETDB1, H3K9me3, H3K14ac, and control read density on SETDB1 overlapping LINE (LINE-SETDB1, blue) and LTR elements (LTR-SETDB1, red). (**b**) Meta-profiles of H3K14ac and control density on all LINE elements (LINE-mm10, blue), SETDB1 overlapping LINEs (LINE-SETDB1, cyan) and LINEs from cluster 6 (LINE-cluster6, yellow). This figure relates to Fig. 6.

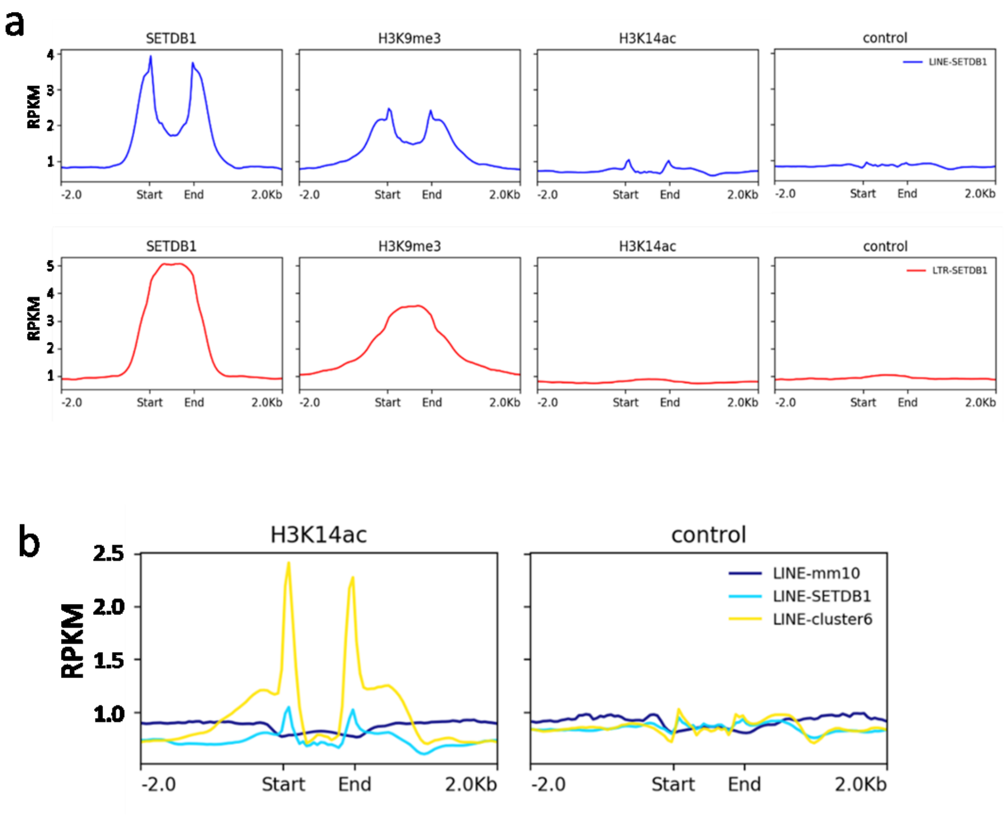

**Supplementary Figure 10: Additional examples of the localization of wildtype and F332A mutant 3TD in mouse cells.** 3TD and F332A were co-expressed as fusion constructs with mCerulean (shown in cyan) or mVenus (shown in yellow) in mouse NIH3T3 cells. While the wt/wt controls show a perfect overlay of the two fluorescence signals, all cells expression wt/F332A combinations showed clear differences between the localization of both fluorophores. Scale bar: 10 µm. Statistics for this experiment are provided in Fig. 7b. This figure relates to Fig. 7.

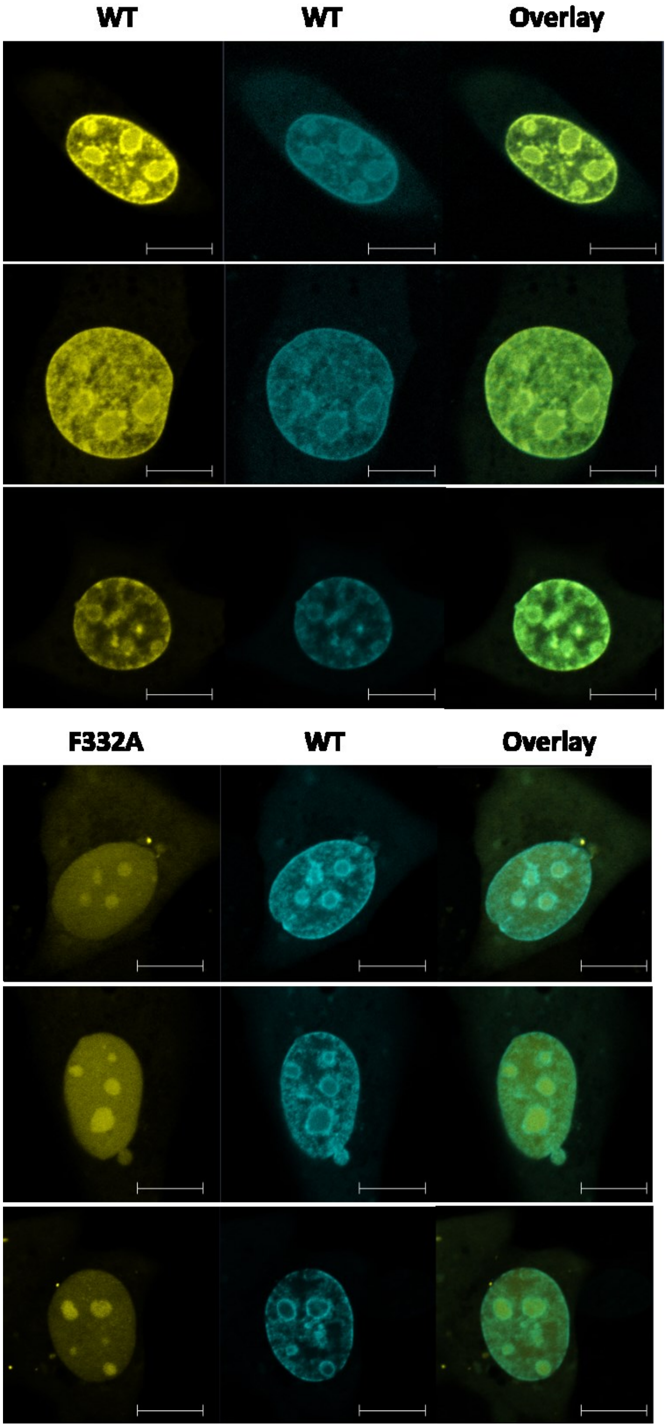

**Supplementary Figure 11: Additional examples of the localization of wildtype and I388A and R394A mutants in mouse cells.** 3TD and mutants were co-expressed as fusion constructs with mCerulean (3TD wildtype, shown in cyan) or mVenus (mutants, shown in yellow) in mouse NIH3T3 cells. All cells expression wt/mutant combinations showed clear differences between the localization of both fluorophores. Scale bar: 10 µm. Statistics for this experiment are provided in Fig. 7b. This figure relates to Fig. 7.

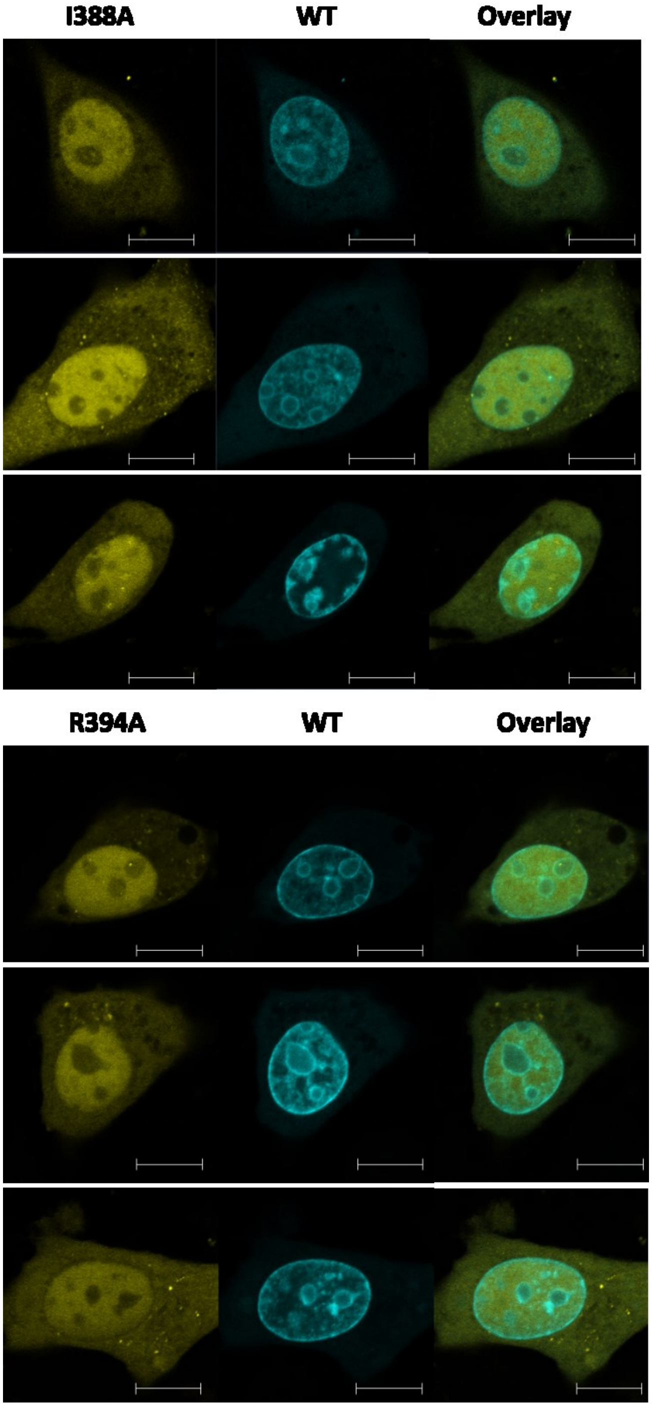

**Supplementary Figure 12: Results of published MS/MS studies of histones isolated from different human cells.** These data document the co-occurrence of H3K14ac with H3K9 methylation[1,2].

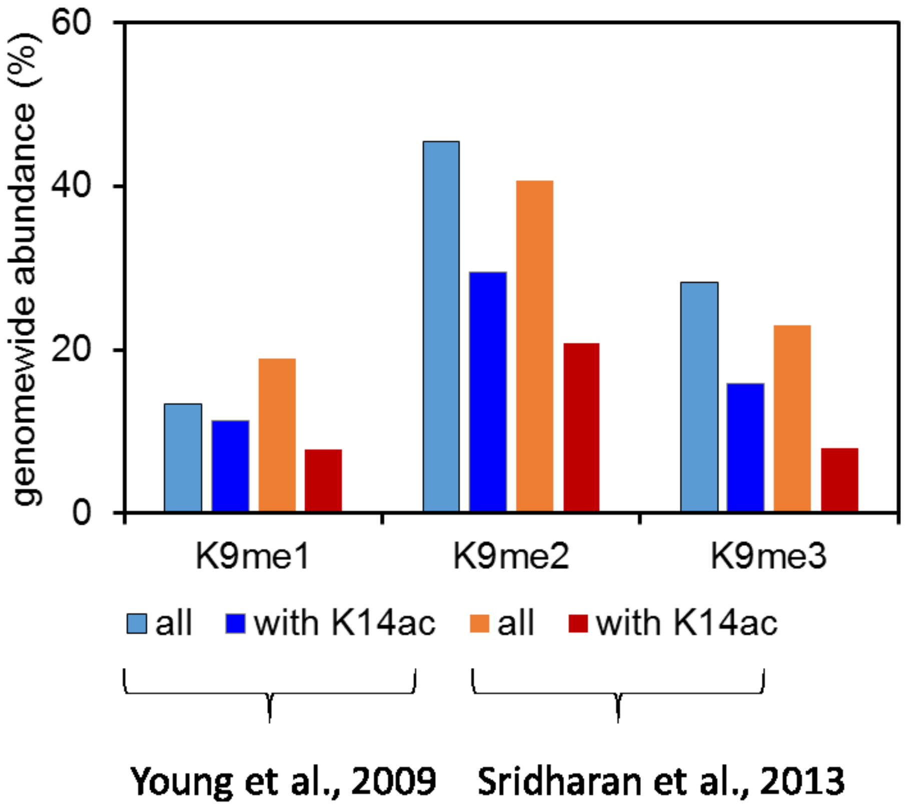

**Supplementary Figure 13: Antibody validation by specificity analysis using modified histone peptide array binding.** The modified histone tail peptide arrays[3] contains two copies of 384 individual Histone tail peptides in different methylation states. Annotations of all spots are provided in Supplementary Table 2. (**a**) Specificity analysis of the Millipore MABE351 H3K14ac antibody. The positions of H3K14ac peptides are indicated. The antibody is highly specific for H3K14ac. (**b**) Specificity analysis of the Abcam ab8898 H3K9me3 antibody. The positions of different trimethylated peptide spots are indicated. The antibody is highly specific for H3K9me3. (**c**) Specificity analysis of the Abcam ab1220 H3K9me2 antibody. The positions of different H3K9 methylated peptide spots are indicated. The antibody is highly specific for H3K9me3. (**d**) Specificity analysis of the Abcam ab8896 H3K9me1 antibody. The positions of different monomethylated peptide spots are indicated. The antibody shows good specificity for H3K9me1. The validation of the anti-H3K4me3 (Abcam ab8580) antibody used in this study is provided in[4]. This figure relates to Fig. 5.

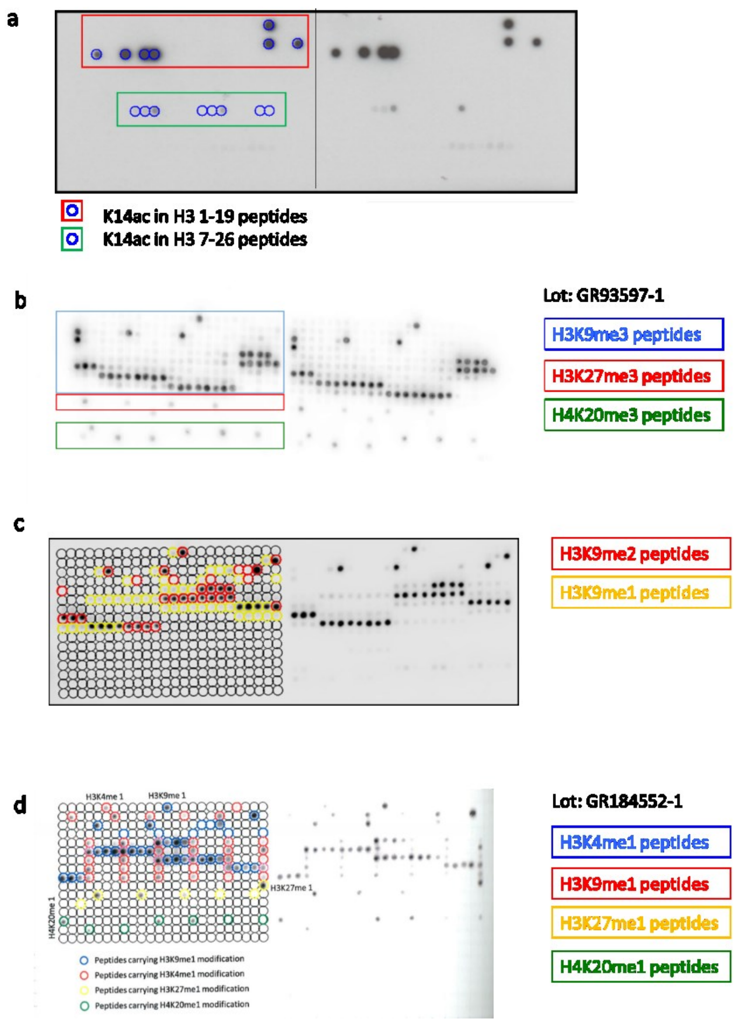

**Supplementary Figure 14: Uncropped pictures of the gel images shown in Fig. 5.**

**Fig. 5a**

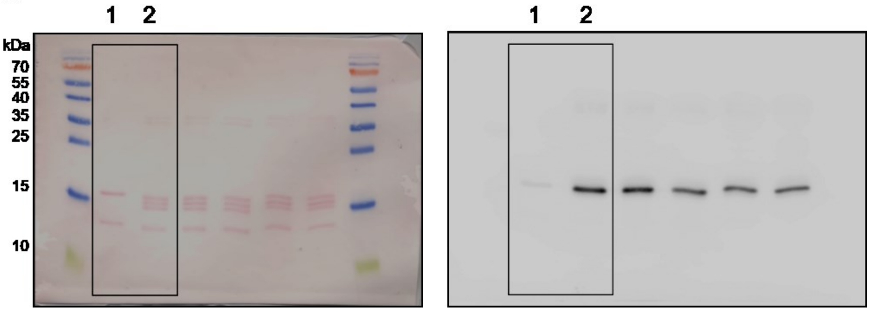


**Fig. 5b**

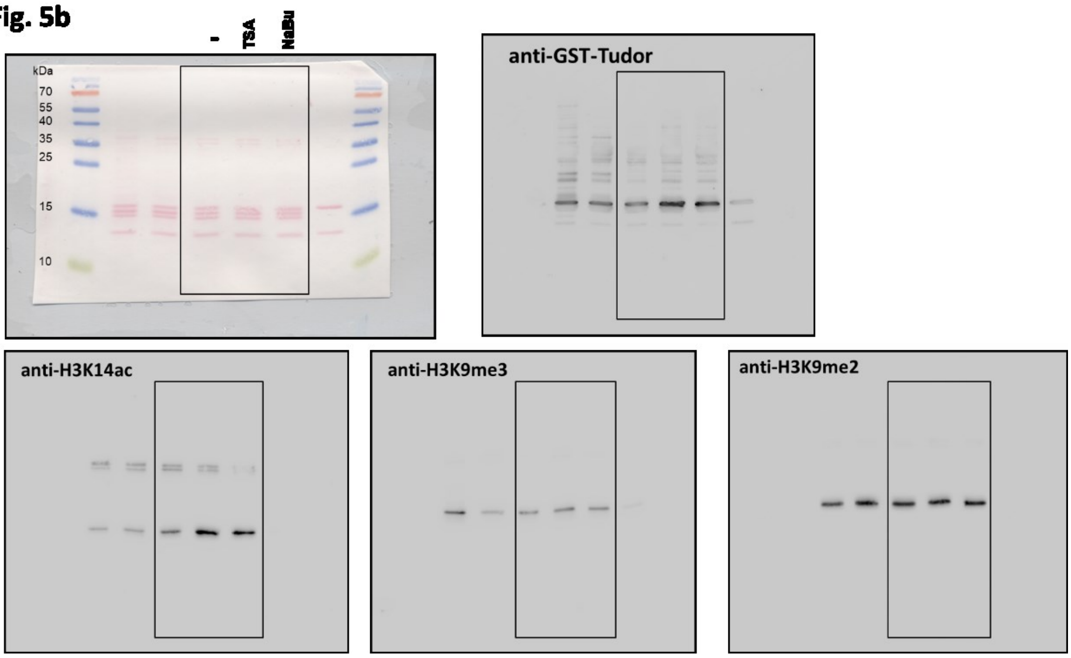


**Fig. 5c**

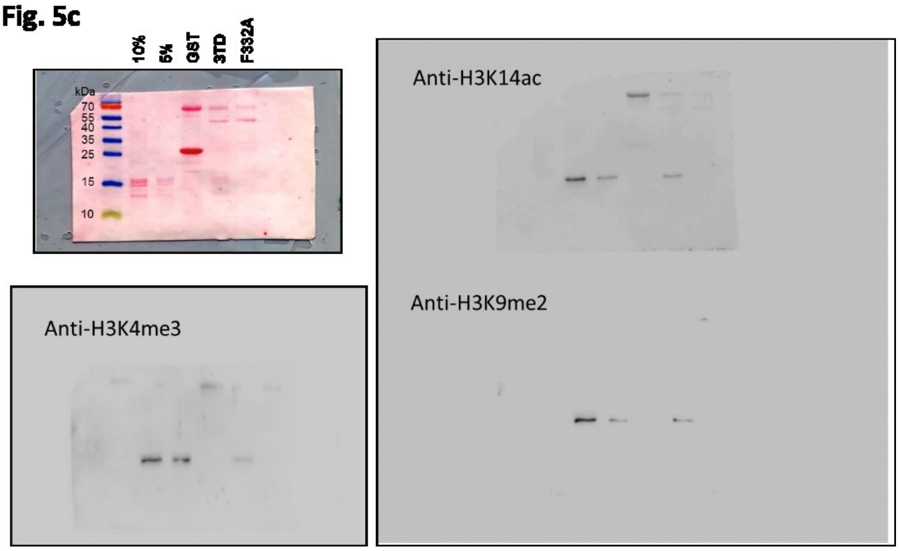

## Supplementary Notes

**Supplementary Note 1: Crystal structure determination.** Diffraction data were collected at APS beam line 19-ID (Structural Biology Center), reduced with XDS[5] and merged with AIMLESS[6], unless otherwise noted. Crystals were kept under cryogenic conditions (100K) during diffraction experiments. Synchrotron sources were tuned to wavelengths between 0.96 and 0.98 Å. For 3TD in complex with K9me3/K14ac, the structure was solved by molecular replacement with SETDB1 coordinates from PDB entry 3DLM (Amaya et al., 2008) and the program PHASER[7]. For 3TD in complex with K9me2/K14ac, preliminary SETDB1 coordinates from the K9me3/K14ac complex were used as a starting model. Difference electron density in the TD2 aromatic cage of this model vaguely resembles a HEPES molecule. For the 3TD in complex with K9me0K14ac, diffraction data were collected at APS beam line 23-ID-B (GM/CA). Preliminary SETDB1 coordinates from the K9me3/K14ac complex were used as a starting model. PHENIX.REFINE[8] was used for automated water peak picking. For the 3TD-W358A in complex with K9me2/K14ac, diffraction data were collected on a copper rotating anode source. Preliminary SETDB1 coordinates from the wildtype K9me2/K14ac complex were used as a starting model. For early refinement steps, diffraction data were reduced with HKL-3000[9]. PHENIX.REFINE was used for automated water peak picking. For the 3TD-W358A in complex with K9me3/K14ac, the structure was solved by molecular replacement with PHASER, using rotating anode data of an additional crystal and atomic coordinates from the wildtype K9me2/K14ac complex. The model was transformed with COOT[10] and CCP4[11] program PDBSET (Evans, 1992) to align it with the other complex models presented here, and further refined against synchrotron data. Models were interactively rebuilt with COOT, refined with REFMAC[12] and validated with PHENIX.MOLPROBITY[13]. In the current models, SETDB1 residue S201 remains an outlier from MOLPROBITY Ramachandran plot contours. Anisotropic displacement parameters were analyzed on the PARVATI[14] server. Deposition in the PDB and publication of the models were prepared with PDB_EXTRACT[15], CCP4 and PHENIX[16] and IOTBX[17].

**Supplementary Note 2: Statistics related to Fig. 6b.** The significance of the overlap of H3K14ac peaks with the intersection of SETDB1 and H3K9me3 peaks shown in Fig. 6b was determined by randomization of the H3K14ac peak regions in the mouse genome (mm10) keeping the number and sizes of the regions constant with the Galaxy ShuffleBed tool. Next, the number of shuffled regions overlapping with the intersection of SETDB1 and H3K9me3 peaks was determined, which represents the expected by-chance overlap. 10 shuffling runs were conducted resulting in 860-940 overlap regions (906.6 ± 24.4). The small standard deviation of the number of overlapping regions in the different shuffled datasets indicates that 10 shuffle rounds are sufficient to draw conclusions. The final p-value for the experimentally observed number of H3K14ac peaks overlapping with the intersection of SETDB1 and H3K9me3 (2231) was determined for a normal distribution using Fishers exact test and found to be highly significant ($p \approx 0$).

| H3K14ac shuffle # | Number of H3K14ac shuffled regions overlapping with SETDB1-H3K9me3 intersection |
|---|---|
| 1 | 897 |
| 2 | 936 |
| 3 | 891 |
| 4 | 929 |
| 5 | 922 |
| 6 | 915 |
| 7 | 886 |
| 8 | 863 |
| 9 | 892 |
| 10 | 935 |
| **average** | **906.6** |
| **stdev** | **24.4** |
| **Observed number of overlapping regions** | **2231** |
| **p-value calculated on normal distribution (Fisher's exact test)** | **0.00E+00** |

**Supplementary Note 3: Statistics related to Fig. 6d.** The analysis for enrichment of the different repeat types in the different clusters by EpiExplorer (Fig. 6d) included repetitions with randomized datasets prepared by EpiExplorer. Based on 10 random repetitions, the average repeat content of each cluster was determined together with the standard error, which represents the statistically expected repeat content of each set of cluster regions. The small standard deviations of the average repeat contents of the randomized samples indicate that the number of randomizations was sufficient to draw conclusions. The p-values for enrichment of the different repeat types in the different clusters were calculated using the average and standard errors from the randomized samples based on a normal distribution by Fisher's exact test.

**Repeat content in clusters 1-8**

| | Cluster 1 | Cluster 2 | Cluster 3 | Cluster 4 | Cluster 5 | Cluster 6 | Cluster 7 | Cluster 8 |
|---|---|---|---|---|---|---|---|---|
| **Any repeat** | 83.3 | 98.7 | 99.2 | 90.9 | 97.4 | 97.7 | 93.0 | 94.3 |
| **LINE** | 17.5 | 23.3 | 23.6 | 40.9 | 17.0 | 88.1 | 48.5 | 37.6 |
| **SINE** | 27.2 | 8.7 | 8.8 | 14.6 | 10.6 | 9.0 | 12.4 | 14.0 |
| **Simple repeats** | 53.5 | 7.0 | 5.5 | 10.0 | 6.3 | 6.4 | 10.7 | 14.0 |
| **LTR** | 25.4 | 95.8 | 95.9 | 47.3 | 91.1 | 17.5 | 46.2 | 58.6 |

**Repeat content in randomized data sets**

| | Average (%) | StDev |
|---|---|---|
| **Any repeat** | 86.8 | 4.8 |
| **LINE** | 39.5 | 5.2 |
| **SINE** | 38.4 | 7.4 |
| **Simple repea** | 28.8 | 6.3 |
| **LTR** | 29.0 | 5.9 |

**p-values for enrichment**

| | Cluster 1 | Cluster 2 | Cluster 3 | Cluster 4 | Cluster 5 | Cluster 6 | Cluster 7 | Cluster 8 |
|---|---|---|---|---|---|---|---|---|
| **Any repeat** | 7.7E-01 | 6.8E-03 | 5.1E-03 | 2.0E-01 | 1.4E-02 | 1.2E-02 | 9.9E-02 | 6.0E-02 |
| **LINE** | 1.0E+00 | 1.0E+00 | 1.0E+00 | 4.0E-01 | 1.0E+00 | 0.0E+00 | 4.1E-02 | 6.5E-01 |
| **SINE** | 9.4E-01 | 1.0E+00 | 1.0E+00 | 1.0E+00 | 1.0E+00 | 1.0E+00 | 1.0E+00 | 1.0E+00 |
| **Simple repeats** | 4.6E-05 | 1.0E+00 | 1.0E+00 | 1.0E+00 | 1.0E+00 | 1.0E+00 | 1.0E+00 | 9.9E-01 |
| **LTR** | 7.3E-01 | 0.0E+00 | 0.0E+00 | 8.8E-04 | 0.0E+00 | 9.7E-01 | 1.6E-03 | 2.2E-07 |

The enrichment of LTRs in clusters 2, 3 and 5 as well as LINEs in cluster 6 (fields shaded in orange) are highly significant.

# Supplementary Tables

**Supplementary Table 1: Data collection and refinement statistics.** Values in parentheses refer to the highest-resolution shell.

| | 3TD-H3K9me2/K14ac | 3TD-H3K9me3/K14ac | 3TD-H3K9me0/K14ac | 3TD(W358A)-H3K9me2/K14ac | 3TD(W358A)-H3K9me3/K14ac |
|---|---|---|---|---|---|
| PDB accession | 6BHD | 6BHE | 6BHG | 6BHH | 6BHI |
| **Data collection** | | | | | |
| Space group | $P2_1$ | $P2_1$ | $P2_1$ | $P2_1$ | $P2_1$ |
| Cell dimensions | | | | | |
| *a*, *b*, *c* (Å) | 37.26,72.45,52.51 | 37.05,71.37,52.05 | 37.71,72.36,52.50 | 37.80,71.80,52.54 | 37.85,71.72,52.69 |
| α, β, γ (°) | 90.00,104.84,90.00 | 90.00,104.23,90.00 | 90.00,104.75,90.00 | 90.00,104.42,90.00 | 90.00,104.48,90.00 |
| Resolution (Å) | 41.57-1.25(1.27-1.25) | 41.20-1.33(1.35-1.33) | 41.56-1.40(1.42-1.40) | 36.61-1.82(1.86-1.82) | 41.57-1.40(1.42-1.40) |
| $R_{merge}$ | 0.050(1.018) | 0.049(1.153) | 0.035(0.826) | 0.121(1.179) | 0.065(0.942) |
| $I / \sigma I$ | 15.1(1.3) | 15.3(1.2) | 19.9(1.4) | 7.9(1.1) | 12.5(1.5) |
| Completeness (%) | 99.8(99.8) | 99.8(99.9) | 99.3(93.1) | 99.8(99.9) | 99.7(100.0) |
| Redundancy | 3.7(3.6) | 3.7(3.6) | 3.7(2.8) | 3.6(3.6) | 3.6(3.7) |
| **Refinement** | | | | | |
| Resolution (Å) | 41.57-1.25 | 35.69-1.35 | 41.56-1.45 | 36.61-1.85 | 41.57-1.40 |
| No. reflections | 70524/3722 | 54617/2872 | 45714/2428 | 22045/1197 | 50707/2671 |
| $R_{work}$ / $R_{free}$ | 0.147/0.183 | 0.135/0.172 | 0.133/0.176 | 0.193/0.237 | 0.148/0.187 |
| No. atoms | 2174 | 2078 | 2069 | 2010 | 2116 |
| Protein | 1843 | 1806 | 1747 | 1699 | 1793 |
| Peptide | 92 | 87 | 59 | 54 | 63 |
| Water | 203 | 149 | 223 | 221 | 225 |
| Other | 36 | 36 | 40 | 36 | 35 |
| *B*-factors | 16.9 | 20.7 | 22.1 | 24.8 | 18.9 |
| Protein | 15.7 | 19.4 | 20.4 | 23.5 | 17.5 |
| Peptide | 17.4 | 20.9 | 21.9 | 24.1 | 19.4 |
| Water | 26.5 | 31.5 | 33.5 | 32.5 | 28.4 |
| Other | 25.5 | 37.5 | 36.1 | 38.0 | 28.1 |
| R.m.s. deviations | | | | | |
| Bond lengths (Å) | 0.019 | 0.017 | 0.016 | 0.013 | 0.017 |
| Bond angles (°) | 1.9 | 1.8 | 1.7 | 1.4 | 1.8 |

**Supplementary Table 2: Full annotation of the peptides present on the modified histone peptide array.** Active Motif, MODified™ Histone Peptide Array, Catalog Nos. 13001 & 13005 [3,18].

| Pos. | Peptide sequence | Name | Mod1 | Mod2 | Mod 3 | Mod 4 | N-terminus |
|---|---|---|---|---|---|---|---|
| A 1 | A R T K Q T A R K S T G G K A P R K Q | H3 1-19 | unmod | | | | free |
| A 2 | A Rme2s T K Q T A R K S T G G K A P R K Q | H3 1-19 | R2me2s | | | | free |
| A 3 | A Rme2a T K Q T A R K S T G G K A P R K Q | H3 1-19 | R2me2a | | | | free |
| A 4 | A Cit T K Q T A R K S T G G K A P R K Q | H3 1-19 | R2Citr | | | | free |
| A 5 | A R pT K Q T A R K S T G G K A P R K Q | H3 1-19 | T3P | | | | free |
| A 6 | A R T Kme1 Q T A R K S T G G K A P R K Q | H3 1-19 | K4me1 | | | | free |
| A 7 | A R T Kme2 Q T A R K S T G G K A P R K Q | H3 1-19 | K4me2 | | | | free |
| A 8 | A R T Kme3 Q T A R K S T G G K A P R K Q | H3 1-19 | K4me3 | | | | free |
| A 9 | A R T Kac Q T A R K S T G G K A P R K Q | H3 1-19 | K4ac | | | | free |
| A10 | A R T K Q T A Rme2s K S T G G K A P R K Q | H3 1-19 | R8me2s | | | | free |
| A11 | A R T K Q T A Rme2a K S T G G K A P R K Q | H3 1-19 | R8me2a | | | | free |
| A12 | A R T K Q T A Cit K S T G G K A P R K Q | H3 1-19 | R8Citr | | | | free |
| A13 | A R T K Q T A R Kme1 S T G G K A P R K Q | H3 1-19 | K9me1 | | | | free |
| A14 | A R T K Q T A R Kme2 S T G G K A P R K Q | H3 1-19 | K9me2 | | | | free |
| A15 | A R T K Q T A R Kme3 S T G G K A P R K Q | H3 1-19 | K9me3 | | | | free |
| A16 | A R T K Q T A R Kac S T G G K A P R K Q | H3 1-19 | K9ac | | | | free |
| A17 | A R T K Q T A R K pS T G G K A P R K Q | H3 1-19 | S10P | | | | free |
| A18 | A R T K Q T A R K S pT G G K A P R K Q | H3 1-19 | T11P | | | | free |
| A19 | A R T K Q T A R K S T G G Kac A P R K Q | H3 1-19 | K14ac | | | | free |

| | | | | | | | |
|---|---|---|---|---|---|---|---|
| A20 | A Rme2s pT K Q T A R K S T G G K A P R K Q | H3 1-19 | R2me2s | T3P | | | free |
| A21 | A Rme2s T Kme1 Q T A R K S T G G K A P R K Q | H3 1-19 | R2me2s | K4me1 | | | free |
| A22 | A Rme2s T Kme2 Q T A R K S T G G K A P R K Q | H3 1-19 | R2me2s | K4me2 | | | free |
| A23 | A Rme2s T Kme3 Q T A R K S T G G K A P R K Q | H3 1-19 | R2me2s | K4me3 | | | free |
| A24 | A Rme2s T Kac Q T A R K S T G G K A P R K Q | H3 1-19 | R2me2s | K4ac | | | free |
| B 1 | A Rme2a pT K Q T A R K S T G G K A P R K Q | H3 1-19 | R2me2a | T3P | | | free |
| B 2 | A Rme2a T Kme1 Q T A R K S T G G K A P R K Q | H3 1-19 | R2me2a | K4me1 | | | free |
| B 3 | A Rme2a T Kme2 Q T A R K S T G G K A P R K Q | H3 1-19 | R2me2a | K4me2 | | | free |
| B 4 | A Rme2a T Kme3 Q T A R K S T G G K A P R K Q | H3 1-19 | R2me2a | K4me3 | | | free |
| B 5 | A Rme2a T Kac Q T A R K S T G G K A P R K Q | H3 1-19 | R2me2a | K4ac | | | free |
| B 6 | A Cit pT K Q T A R K S T G G K A P R K Q | H3 1-19 | R2Citr | T3P | | | free |
| B 7 | A Cit T Kme1 Q T A R K S T G G K A P R K Q | H3 1-19 | R2Citr | K4me1 | | | free |
| B 8 | A Cit T Kme2 Q T A R K S T G G K A P R K Q | H3 1-19 | R2Citr | K4me2 | | | free |
| B 9 | A Cit T Kme3 Q T A R K S T G G K A P R K Q | H3 1-19 | R2Citr | K4me3 | | | free |
| B10 | A Cit T Kac Q T A R K S T G G K A P R K Q | H3 1-19 | R2Citr | K4ac | | | free |
| B11 | A R pT Kme1 Q T A R K S T G G K A P R K Q | H3 1-19 | T3P | K4me1 | | | free |
| B12 | A R pT Kme2 Q T A R K S T G G K A P R K Q | H3 1-19 | T3P | K4me2 | | | free |
| B13 | A R pT Kme3 Q T A R K S T G G K A P R K Q | H3 1-19 | T3P | K4me3 | | | free |
| B14 | A R pT Kac Q T A R K S T G G K A P R K Q | H3 1-19 | T3P | K4ac | | | free |
| B15 | A Rme2s pT Kme1 Q T A R K S T G G K A P R K Q | H3 1-19 | R2me2s | T3P | K4me 1 | | free |
| B16 | A Rme2s pT Kme2 Q T A R K S T G G K A P R K Q | H3 1-19 | R2me2s | T3P | K4me 2 | | free |
| B17 | A Rme2s pT Kme3 Q T A R K S T G G K A P R K Q | H3 1-19 | R2me2s | T3P | K4me 3 | | free |

| | | | | | | | |
|---|---|---|---|---|---|---|---|
| B18 | A Rme2s pT Kac Q T A R K S T G G K A P R K Q | H3 1-19 | R2me2s | T3P | K4ac | | free |
| B19 | A Rme2a pT Kme1 Q T A R K S T G G K A P R K Q | H3 1-19 | R2me2a | T3P | K4me 1 | | free |
| B20 | A Rme2a pT Kme2 Q T A R K S T G G K A P R K Q | H3 1-19 | R2me2a | T3P | K4me 2 | | free |
| B21 | A Rme2a pT Kme3 Q T A R K S T G G K A P R K Q | H3 1-19 | R2me2a | T3P | K4me 3 | | free |
| B22 | A Rme2a pT Kac Q T A R K S T G G K A P R K Q | H3 1-19 | R2me2a | T3P | K4ac | | free |
| B23 | A R T K Q T A Rme2a Kme1 S T G G K A P R K Q | H3 1-19 | R8me2s | K9me1 | | | free |
| B24 | A R T K Q T A Rme2a Kme2 S T G G K A P R K Q | H3 1-19 | R8me2s | K9me2 | | | free |
| C 1 | A R T K Q T A Rme2a Kme3 S T G G K A P R K Q | H3 1-19 | R8me2s | K9me3 | | | free |
| C 2 | A R T K Q T A Rme2a Kac S T G G K A P R K Q | H3 1-19 | R8me2s | K9ac | | | free |
| C 3 | A R T K Q T A Rme2a K pS T G G K A P R K Q | H3 1-19 | R8me2s | S10P | | | free |
| C 4 | A R T K Q T A Rme2a K S pT G G K A P R K Q | H3 1-19 | R8me2s | T11P | | | free |
| C 5 | A R T K Q T A Rme2a Kme1 S T G G K A P R K Q | H3 1-19 | R8me2a | K9me1 | | | free |
| C 6 | A R T K Q T A Rme2a Kme2 S T G G K A P R K Q | H3 1-19 | R8me2a | K9me2 | | | free |
| C 7 | A R T K Q T A Rme2a Kme3 S T G G K A P R K Q | H3 1-19 | R8me2a | K9me3 | | | free |
| C 8 | A R T K Q T A Rme2a Kac S T G G K A P R K Q | H3 1-19 | R8me2a | K9ac | | | free |
| C 9 | A R T K Q T A Rme2a K pS T G G K A P R K Q | H3 1-19 | R8me2a | S10P | | | free |
| C10 | A R T K Q T A Rme2a K S pT G G K A P R K Q | H3 1-19 | R8me2a | T11P | | | free |
| C11 | A R T K Q T A Cit Kme1 S T G G K A P R K Q | H3 1-19 | R8Citr | K9me1 | | | free |
| C12 | A R T K Q T A Cit Kme2 S T G G K A P R K Q | H3 1-19 | R8Citr | K9me2 | | | free |
| C13 | A R T K Q T A Cit Kme3 S T G G K A P R K Q | H3 1-19 | R8Citr | K9me3 | | | free |
| C14 | A R T K Q T A Cit Kac S T G G K A P R K Q | H3 1-19 | R8Citr | K9ac | | | free |
| C15 | A R T K Q T A Cit K pS T G G K A P R K Q | H3 1-19 | R8Citr | S10P | | | free |

| | | | | | | | |
|---|---|---|---|---|---|---|---|
| C16 | A R T K Q T A Cit K S pT G G K A P R K Q | H3 1-19 | R8Citr | T11P | | | free |
| C17 | A R T K Q T A R Kme1 pS T G G K A P R K Q | H3 1-19 | K9me1 | S10P | | | free |
| C18 | A R T K Q T A R Kme1 S pT G G K A P R K Q | H3 1-19 | K9me1 | T11P | | | free |
| C19 | A R T K Q T A R Kme1 S T G G Kac A P R K Q | H3 1-19 | K9me1 | K14ac | | | free |
| C20 | A R T K Q T A R Kme2 pS T G G K A P R K Q | H3 1-19 | K9me2 | S10P | | | free |
| C21 | A R T K Q T A R Kme2 S pT G G K A P R K Q | H3 1-19 | K9me2 | T11P | | | free |
| C22 | A R T K Q T A R Kme2 S T G G Kac A P R K Q | H3 1-19 | K9me2 | K14ac | | | free |
| C23 | A R T K Q T A R Kme3 pS T G G K A P R K Q | H3 1-19 | K9me3 | S10P | | | free |
| C24 | A R T K Q T A R Kme3 S pT G G K A P R K Q | H3 1-19 | K9me3 | T11P | | | free |
| D 1 | A R T K Q T A R Kme3 S T G G Kac A P R K Q | H3 1-19 | K9me3 | K14ac | | | free |
| D 2 | A R T K Q T A R Kac pS T G G K A P R K Q | H3 1-19 | K9ac | S10P | | | free |
| D 3 | A R T K Q T A R Kac S pT G G K A P R K Q | H3 1-19 | K9ac | T11P | | | free |
| D 4 | A R T K Q T A R Kac S T G G Kac A P R K Q | H3 1-19 | K9ac | K14ac | | | free |
| D 5 | A R T K Q T A R K pS pT G G K A P R K Q | H3 1-19 | S10P | T11P | | | free |
| D 6 | A R T K Q T A R K pS T G G Kac A P R K Q | H3 1-19 | S10P | K14ac | | | free |
| D 7 | A R T K Q T A R K S pT G G Kac A P R K Q | H3 1-19 | T11P | K14ac | | | free |
| D 8 | A R T K Q T A Rme2s Kme1 pS T G G K A P R K Q | H3 1-19 | R8me2s | K9me1 | S10P | | free |
| D 9 | A R T K Q T A Rme2s Kme2 pS T G G K A P R K Q | H3 1-19 | R8me2s | K9me2 | S10P | | free |
| D10 | A R T K Q T A Rme2s Kme3 pS T G G K A P R K Q | H3 1-19 | R8me2s | K9me3 | S10P | | free |
| D11 | A R T K Q T A Rme2s Kac pS T G G K A P R K Q | H3 1-19 | R8me2s | K9ac | S10P | | free |
| D12 | A R T K Q T A Rme2s Kme1 S pT G G K A P R K Q | H3 1-19 | R8me2s | K9me1 | T11P | | free |
| D13 | A R T K Q T A Rme2s Kme2 S pT G G K A P R K Q | H3 1-19 | R8me2s | K9me2 | T11P | | free |

| | | | | | | | |
|---|---|---|---|---|---|---|---|
| D14 | A R T K Q T A Rme2s Kme3 S pT G G K A P R K Q | H3 1-19 | R8me2s | K9me3 | T11P | | free |
| D15 | A R T K Q T A Rme2s Kac S pT G G K A P R K Q | H3 1-19 | R8me2s | K9ac | T11P | | free |
| D16 | A R T K Q T A Rme2a Kme1 pS T G G K A P R K Q | H3 1-19 | R8me2a | K9me1 | S10P | | free |
| D17 | A R T K Q T A Rme2a Kme2 pS T G G K A P R K Q | H3 1-19 | R8me2a | K9me2 | S10P | | free |
| D18 | A R T K Q T A Rme2a Kme3 pS T G G K A P R K Q | H3 1-19 | R8me2a | K9me3 | S10P | | free |
| D19 | A R T K Q T A Rme2a Kac pS T G G K A P R K Q | H3 1-19 | R8me2a | K9ac | S10P | | free |
| D20 | A R T K Q T A Rme2a Kme1 S pT G G K A P R K Q | H3 1-19 | R8me2a | K9me1 | T11P | | free |
| D21 | A R T K Q T A Rme2a Kme2 S pT G G K A P R K Q | H3 1-19 | R8me2a | K9me2 | T11P | | free |
| D22 | A R T K Q T A Rme2a Kme3 S pT G G K A P R K Q | H3 1-19 | R8me2a | K9me3 | T11P | | free |
| D23 | A R T K Q T A Rme2a Kac S pT G G K A P R K Q | H3 1-19 | R8me2a | K9ac | T11P | | free |
| D24 | A R T K Q T A Rme2a Kme1 pS pT G G K A P R K Q | H3 1-19 | R8me2a | K9me1 | S10P | T11P | free |
| E 1 | A R T K Q T A Rme2a Kme2 pS pT G G K A P R K Q | H3 1-19 | R8me2a | K9me2 | S10P | T11P | free |
| E 2 | A R T K Q T A Rme2a Kme3 pS pT G G K A P R K Q | H3 1-19 | R8me2a | K9me3 | S10P | T11P | free |
| E 3 | A R T K Q T A Rme2a Kac pS pT G G K A P R K Q | H3 1-19 | R8me2a | K9ac | S10P | T11P | free |
| E 4 | A Rme2s T Kme1 Q T A Rme2s K S T G G K A P R K Q | H3 1-19 | R2me2s | K4me1 | R8me 2s | | free |
| E 5 | A Rme2s T Kme2 Q T A Rme2s K S T G G K A P R K Q | H3 1-19 | R2me2s | K4me2 | R8me 2s | | free |
| E 6 | A Rme2s T Kme3 Q T A Rme2s K S T G G K A P R K Q | H3 1-19 | R2me2s | K4me3 | R8me 2s | | free |
| E 7 | A Rme2s T Kac Q T A Rme2s K S T G G K A P R K Q | H3 1-19 | R2me2s | K4ac | R8me 2s | | free |
| E 8 | A Rme2a T Kme1 Q T A Rme2a K S T G G K A P R K Q | H3 1-19 | R2me2a | K4me1 | R8me 2a | | free |
| E 9 | A Rme2a T Kme2 Q T A Rme2a K S T G G K A P R K Q | H3 1-19 | R2me2a | K4me2 | R8me 2a | | free |
| E10 | A Rme2a T Kme3 Q T A Rme2a K S T G G K A P R K Q | H3 1-19 | R2me2a | K4me3 | R8me 2a | | free |
| E11 | A Rme2a T Kac Q T A Rme2a K S T G G K A P R K Q | H3 1-19 | R2me2a | K4ac | R8me 2a | | free |

| | | | | | | | |
|---|---|---|---|---|---|---|---|
| E12 | A Rme2s T Kme1 Q T A R Kme1 S T G G K A P R K Q | H3 1-19 | R2me2s | K4me1 | K9me 1 | | free |
| E13 | A Rme2s T Kme2 Q T A R Kme1 S T G G K A P R K Q | H3 1-19 | R2me2s | K4me2 | K9me 1 | | free |
| E14 | A Rme2s T Kme3 Q T A R Kme1 S T G G K A P R K Q | H3 1-19 | R2me2s | K4me3 | K9me 1 | | free |
| E15 | A Rme2s T Kac Q T A R Kme1 S T G G K A P R K Q | H3 1-19 | R2me2s | K4ac | K9me 1 | | free |
| E16 | A Rme2a T Kme1 Q T A R Kme2 S T G G K A P R K Q | H3 1-19 | R2me2a | K4me1 | K9me 2 | | free |
| E17 | A Rme2a T Kme2 Q T A R Kme2 S T G G K A P R K Q | H3 1-19 | R2me2a | K4me2 | K9me 2 | | free |
| E18 | A Rme2a T Kme3 Q T A R Kme2 S T G G K A P R K Q | H3 1-19 | R2me2a | K4me3 | K9me 2 | | free |
| E19 | A Rme2a T Kac Q T A R Kme2 S T G G K A P R K Q | H3 1-19 | R2me2a | K4ac | K9me 2 | | free |
| E20 | A Rme2s T Kme1 Q T A R Kme3 S T G G K A P R K Q | H3 1-19 | R2me2s | K4me1 | K9me 3 | | free |
| E21 | A Rme2s T Kme2 Q T A R Kme3 S T G G K A P R K Q | H3 1-19 | R2me2s | K4me2 | K9me 3 | | free |
| E22 | A Rme2s T Kme3 Q T A R Kme3 S T G G K A P R K Q | H3 1-19 | R2me2s | K4me3 | K9me 3 | | free |
| E23 | A Rme2s T Kac Q T A R Kme3 S T G G K A P R K Q | H3 1-19 | R2me2s | K4ac | K9me 3 | | free |
| E24 | A Rme2a T Kme1 Q T A R Kac S T G G K A P R K Q | H3 1-19 | R2me2a | K4me1 | K9ac | | free |
| F 1 | A Rme2a T Kme2 Q T A R Kac S T G G K A P R K Q | H3 1-19 | R2me2a | K4me2 | K9ac | | free |
| F 2 | A Rme2a T Kme3 Q T A R Kac S T G G K A P R K Q | H3 1-19 | R2me2a | K4me3 | K9ac | | free |
| F 3 | A Rme2a T Kac Q T A R Kac S T G G K A P R K Q | H3 1-19 | R2me2a | K4ac | K9ac | | free |
| F 4 | A R T Kme1 Q T A Rme2s Kme1 S T G G K A P R K Q | H3 1-19 | K4me1 | R8me2 s | K9me 1 | | free |
| F 5 | A R T Kme2 Q T A Rme2s Kme1 S T G G K A P R K Q | H3 1-19 | K4me2 | R8me2 s | K9me 1 | | free |
| F 6 | A R T Kme3 Q T A Rme2s Kme1 S T G G K A P R K Q | H3 1-19 | K4me3 | R8me2 s | K9me 1 | | free |
| F 7 | A R T Kac Q T A Rme2s Kme1 S T G G K A P R K Q | H3 1-19 | K4ac | R8me2 s | K9me 1 | | free |
| F 8 | A R T Kme1 Q T A Rme2a Kme1 S T G G K A P R K Q | H3 1-19 | K4me1 | R8me2 a | K9me 1 | | free |
| F 9 | A R T Kme2 Q T A Rme2a Kme1 S T G G K A P R K Q | H3 1-19 | K4me2 | R8me2 a | K9me 1 | | free |

| | | | | | | | |
|---|---|---|---|---|---|---|---|
| F10 | A R T Kme3 Q T A Rme2a Kme1 S T G G K A P R K Q | H3 1-19 | K4me3 | R8me2a | K9me1 | | free |
| F11 | A R T Kac Q T A Rme2a Kme1 S T G G K A P R K Q | H3 1-19 | K4ac | R8me2a | K9me1 | | free |
| F12 | A R T Kme1 Q T A Rme2s Kme2 S T G G K A P R K Q | H3 1-19 | K4me1 | R8me2s | K9me2 | | free |
| F13 | A R T Kme2 Q T A Rme2s Kme2 S T G G K A P R K Q | H3 1-19 | K4me2 | R8me2s | K9me2 | | free |
| F14 | A R T Kme3 Q T A Rme2s Kme2 S T G G K A P R K Q | H3 1-19 | K4me3 | R8me2s | K9me2 | | free |
| F15 | A R T Kac Q T A Rme2s Kme2 S T G G K A P R K Q | H3 1-19 | K4ac | R8me2s | K9me2 | | free |
| F16 | A R T Kme1 Q T A Rme2a Kme2 S T G G K A P R K Q | H3 1-19 | K4me1 | R8me2a | K9me2 | | free |
| F17 | A R T Kme2 Q T A Rme2a Kme2 S T G G K A P R K Q | H3 1-19 | K4me2 | R8me2a | K9me2 | | free |
| F18 | A R T Kme3 Q T A Rme2a Kme2 S T G G K A P R K Q | H3 1-19 | K4me3 | R8me2a | K9me2 | | free |
| F19 | A R T Kac Q T A Rme2a Kme2 S T G G K A P R K Q | H3 1-19 | K4ac | R8me2a | K9me2 | | free |
| F20 | A R T Kme1 Q T A Rme2s Kme3 S T G G K A P R K Q | H3 1-19 | K4me1 | R8me2s | K9me3 | | free |
| F21 | A R T Kme2 Q T A Rme2s Kme3 S T G G K A P R K Q | H3 1-19 | K4me2 | R8me2s | K9me3 | | free |
| F22 | A R T Kme3 Q T A Rme2s Kme3 S T G G K A P R K Q | H3 1-19 | K4me3 | R8me2s | K9me3 | | free |
| F23 | A R T Kac Q T A Rme2s Kme3 S T G G K A P R K Q | H3 1-19 | K4ac | R8me2s | K9me3 | | free |
| F24 | A R T Kme1 Q T A Rme2a Kme3 S T G G K A P R K Q | H3 1-19 | K4me1 | R8me2a | K9me3 | | free |
| G 1 | A R T Kme2 Q T A Rme2a Kme3 S T G G K A P R K Q | H3 1-19 | K4me2 | R8me2a | K9me3 | | free |
| G 2 | A R T Kme3 Q T A Rme2a Kme3 S T G G K A P R K Q | H3 1-19 | K4me3 | R8me2a | K9me3 | | free |
| G 3 | A R T Kac Q T A Rme2a Kme3 S T G G K A P R K Q | H3 1-19 | K4ac | R8me2a | K9me3 | | free |
| G 4 | A R T Kme1 Q T A Rme2s Kac S T G G K A P R K Q | H3 1-19 | K4me1 | R8me2s | K9ac | | free |
| G 5 | A R T Kme2 Q T A Rme2s Kac S T G G K A P R K Q | H3 1-19 | K4me2 | R8me2s | K9ac | | free |
| G 6 | A R T Kme3 Q T A Rme2s Kac S T G G K A P R K Q | H3 1-19 | K4me3 | R8me2s | K9ac | | free |
| G 7 | A R T Kac Q T A Rme2s Kac S T G G K A P R K Q | H3 1-19 | K4ac | R8me2s | K9ac | | free |

| | | | | | | | |
|---|---|---|---|---|---|---|---|
| G 8 | A R T Kme1 Q T A Rme2a Kac S T G G K A P R K Q | H3 1-19 | K4me1 | R8me2a | K9ac | | free |
| G 9 | A R T Kme2 Q T A Rme2a Kac S T G G K A P R K Q | H3 1-19 | K4me2 | R8me2a | K9ac | | free |
| G10 | A R T Kme3 Q T A Rme2a Kac S T G G K A P R K Q | H3 1-19 | K4me3 | R8me2a | K9ac | | free |
| G11 | A R T Kac Q T A Rme2a Kac S T G G K A P R K Q | H3 1-19 | K4ac | R8me2a | K9ac | | free |
| G12 | A Rme2s T Kme1 Q T A Rme2s Kme1 S T G G K A P R K Q | H3 1-19 | R2me2s | K4me1 | R8me2s | K9me1 | free |
| G13 | A Rme2s T Kme2 Q T A Rme2s Kme1 S T G G K A P R K Q | H3 1-19 | R2me2s | K4me2 | R8me2s | K9me1 | free |
| G14 | A Rme2s T Kme3 Q T A Rme2s Kme1 S T G G K A P R K Q | H3 1-19 | R2me2s | K4me3 | R8me2s | K9me1 | free |
| G15 | A Rme2s T Kac Q T A Rme2s Kme1 S T G G K A P R K Q | H3 1-19 | R2me2s | K4ac | R8me2s | K9me1 | free |
| G16 | A Rme2a T Kme1 Q T A Rme2s Kme1 S T G G K A P R K Q | H3 1-19 | R2me2a | K4me1 | R8me2s | K9me1 | free |
| G17 | A Rme2a T Kme2 Q T A Rme2s Kme1 S T G G K A P R K Q | H3 1-19 | R2me2a | K4me2 | R8me2s | K9me1 | free |
| G18 | A Rme2a T Kme3 Q T A Rme2s Kme1 S T G G K A P R K Q | H3 1-19 | R2me2a | K4me3 | R8me2s | K9me1 | free |
| G19 | A Rme2a T Kac Q T A Rme2s Kme1 S T G G K A P R K Q | H3 1-19 | R2me2a | K4ac | R8me2s | K9me1 | free |
| G20 | A Rme2s T Kme1 Q T A Rme2s Kme2 S T G G K A P R K Q | H3 1-19 | R2me2s | K4me1 | R8me2s | K9me2 | free |
| G21 | A Rme2s T Kme2 Q T A Rme2s Kme2 S T G G K A P R K Q | H3 1-19 | R2me2s | K4me2 | R8me2s | K9me2 | free |
| G22 | A Rme2s T Kme3 Q T A Rme2s Kme2 S T G G K A P R K Q | H3 1-19 | R2me2s | K4me3 | R8me2s | K9me2 | free |
| G23 | A Rme2s T Kac Q T A Rme2s Kme2 S T G G K A P R K Q | H3 1-19 | R2me2s | K4ac | R8me2s | K9me2 | free |
| G24 | A Rme2a T Kme1 Q T A Rme2s Kme2 S T G G K A P R K Q | H3 1-19 | R2me2a | K4me1 | R8me2s | K9me2 | free |
| H 1 | A Rme2a T Kme2 Q T A Rme2s Kme2 S T G G K A P R K Q | H3 1-19 | R2me2a | K4me2 | R8me2s | K9me2 | free |
| H 2 | A Rme2a T Kme3 Q T A Rme2s Kme2 S T G G K A P R K Q | H3 1-19 | R2me2a | K4me3 | R8me2s | K9me2 | free |
| H 3 | A Rme2a T Kac Q T A Rme2s Kme2 S T G G K A P R K Q | H3 1-19 | R2me2a | K4ac | R8me2s | K9me2 | free |
| H 4 | A Rme2s T Kme1 Q T A Rme2s Kme3 S T G G K A P R K Q | H3 1-19 | R2me2s | K4me1 | R8me2s | K9me3 | free |
| H 5 | A Rme2s T Kme2 Q T A Rme2s Kme3 S T G G K A P R K Q | H3 1-19 | R2me2s | K4me2 | R8me2s | K9me3 | free |

| | | | | | | | |
|---|---|---|---|---|---|---|---|
| H 6 | A Rme2s T Kme3 Q T A Rme2s Kme3 S T G G K A P R K Q | H3 1-19 | R2me2s | K4me3 | R8me 2s | K9me3 | free |
| H 7 | A Rme2s T Kac Q T A Rme2s Kme3 S T G G K A P R K Q | H3 1-19 | R2me2s | K4ac | R8me 2s | K9me3 | free |
| H 8 | A Rme2a T Kme1 Q T A Rme2s Kme3 S T G G K A P R K Q | H3 1-19 | R2me2a | K4me1 | R8me 2s | K9me3 | free |
| H 9 | A Rme2a T Kme2 Q T A Rme2s Kme3 S T G G K A P R K Q | H3 1-19 | R2me2a | K4me2 | R8me 2s | K9me3 | free |
| H10 | A Rme2a T Kme3 Q T A Rme2s Kme3 S T G G K A P R K Q | H3 1-19 | R2me2a | K4me3 | R8me 2s | K9me3 | free |
| H11 | A Rme2a T Kac Q T A Rme2s Kme3 S T G G K A P R K Q | H3 1-19 | R2me2a | K4ac | R8me 2s | K9me3 | free |
| H12 | A Rme2s T Kme1 Q T A Rme2s Kac S T G G K A P R K Q | H3 1-19 | R2me2s | K4me1 | R8me 2s | K9ac | free |
| H13 | A Rme2s T Kme2 Q T A Rme2s Kac S T G G K A P R K Q | H3 1-19 | R2me2s | K4me2 | R8me 2s | K9ac | free |
| H14 | A Rme2s T Kme3 Q T A Rme2s Kac S T G G K A P R K Q | H3 1-19 | R2me2s | K4me3 | R8me 2s | K9ac | free |
| H15 | A Rme2s T Kac Q T A Rme2s Kac S T G G K A P R K Q | H3 1-19 | R2me2s | K4ac | R8me 2s | K9ac | free |
| H16 | A Rme2a T Kme1 Q T A Rme2s Kac S T G G K A P R K Q | H3 1-19 | R2me2a | K4me1 | R8me 2s | K9ac | free |
| H17 | A Rme2a T Kme2 Q T A Rme2s Kac S T G G K A P R K Q | H3 1-19 | R2me2a | K4me2 | R8me 2s | K9ac | free |
| H18 | A Rme2a T Kme3 Q T A Rme2s Kac S T G G K A P R K Q | H3 1-19 | R2me2a | K4me3 | R8me 2s | K9ac | free |
| H19 | A Rme2a T Kac Q T A Rme2s Kac S T G G K A P R K Q | H3 1-19 | R2me2a | K4ac | R8me 2s | K9ac | free |
| H20 | A Rme2s T Kme1 Q T A Rme2a Kme1 S T G G K A P R K Q | H3 1-19 | R2me2s | K4me1 | R8me 2a | K9me1 | free |
| H21 | A Rme2s T Kme2 Q T A Rme2a Kme1 S T G G K A P R K Q | H3 1-19 | R2me2s | K4me2 | R8me 2a | K9me1 | free |
| H22 | A Rme2s T Kme3 Q T A Rme2a Kme1 S T G G K A P R K Q | H3 1-19 | R2me2s | K4me3 | R8me 2a | K9me1 | free |
| H23 | A Rme2s T Kac Q T A Rme2a Kme1 S T G G K A P R K Q | H3 1-19 | R2me2s | K4ac | R8me 2a | K9me1 | free |
| H24 | A Rme2a T Kme1 Q T A Rme2a Kme1 S T G G K A P R K Q | H3 1-19 | R2me2a | K4me1 | R8me 2a | K9me1 | free |
| I 1 | A Rme2a T Kme2 Q T A Rme2a Kme1 S T G G K A P R K Q | H3 1-19 | R2me2a | K4me2 | R8me 2a | K9me1 | free |
| I 2 | A Rme2a T Kme3 Q T A Rme2a Kme1 S T G G K A P R K Q | H3 1-19 | R2me2a | K4me3 | R8me 2a | K9me1 | free |
| I 3 | A Rme2a T Kac Q T A Rme2a Kme1 S T G G K A P R K Q | H3 1-19 | R2me2a | K4ac | R8me 2a | K9me1 | free |

| | | | | | | | |
|---|---|---|---|---|---|---|---|
| I 4 | A Rme2s T Kme1 Q T A Rme2a Kme2 S T G G K A P R K Q | H3 1-19 | R2me2s | K4me1 | R8me 2a | K9me2 | free |
| I 5 | A Rme2s T Kme2 Q T A Rme2a Kme2 S T G G K A P R K Q | H3 1-19 | R2me2s | K4me2 | R8me 2a | K9me2 | free |
| I 6 | A Rme2s T Kme3 Q T A Rme2a Kme2 S T G G K A P R K Q | H3 1-19 | R2me2s | K4me3 | R8me 2a | K9me2 | free |
| I 7 | A Rme2s T Kac Q T A Rme2a Kme2 S T G G K A P R K Q | H3 1-19 | R2me2s | K4ac | R8me 2a | K9me2 | free |
| I 8 | A Rme2a T Kme1 Q T A Rme2a Kme2 S T G G K A P R K Q | H3 1-19 | R2me2a | K4me1 | R8me 2a | K9me2 | free |
| I 9 | A Rme2a T Kme2 Q T A Rme2a Kme2 S T G G K A P R K Q | H3 1-19 | R2me2a | K4me2 | R8me 2a | K9me2 | free |
| I10 | A Rme2a T Kme3 Q T A Rme2a Kme2 S T G G K A P R K Q | H3 1-19 | R2me2a | K4me3 | R8me 2a | K9me2 | free |
| I11 | A Rme2a T Kac Q T A Rme2a Kme2 S T G G K A P R K Q | H3 1-19 | R2me2a | K4ac | R8me 2a | K9me2 | free |
| I12 | A Rme2s T Kme1 Q T A Rme2a Kme3 S T G G K A P R K Q | H3 1-19 | R2me2s | K4me1 | R8me 2a | K9me3 | free |
| I13 | A Rme2s T Kme2 Q T A Rme2a Kme3 S T G G K A P R K Q | H3 1-19 | R2me2s | K4me2 | R8me 2a | K9me3 | free |
| I14 | A Rme2s T Kme3 Q T A Rme2a Kme3 S T G G K A P R K Q | H3 1-19 | R2me2s | K4me3 | R8me 2a | K9me3 | free |
| I15 | A Rme2s T Kac Q T A Rme2a Kme3 S T G G K A P R K Q | H3 1-19 | R2me2s | K4ac | R8me 2a | K9me3 | free |
| I16 | A Rme2a T Kme1 Q T A Rme2a Kme3 S T G G K A P R K Q | H3 1-19 | R2me2a | K4me1 | R8me 2a | K9me3 | free |
| I17 | A Rme2a T Kme2 Q T A Rme2a Kme3 S T G G K A P R K Q | H3 1-19 | R2me2a | K4me2 | R8me 2a | K9me3 | free |
| I18 | A Rme2a T Kme3 Q T A Rme2a Kme3 S T G G K A P R K Q | H3 1-19 | R2me2a | K4me3 | R8me 2a | K9me3 | free |
| I19 | A Rme2a T Kac Q T A Rme2a Kme3 S T G G K A P R K Q | H3 1-19 | R2me2a | K4ac | R8me 2a | K9me3 | free |
| I20 | A Rme2s T Kme1 Q T A Rme2a Kac S T G G K A P R K Q | H3 1-19 | R2me2s | K4me1 | R8me 2a | K9ac | free |
| I21 | A Rme2s T Kme2 Q T A Rme2a Kac S T G G K A P R K Q | H3 1-19 | R2me2s | K4me2 | R8me 2a | K9ac | free |
| I22 | A Rme2s T Kme3 Q T A Rme2a Kac S T G G K A P R K Q | H3 1-19 | R2me2s | K4me3 | R8me 2a | K9ac | free |
| I23 | A Rme2s T Kac Q T A Rme2a Kac S T G G K A P R K Q | H3 1-19 | R2me2s | K4ac | R8me 2a | K9ac | free |
| I24 | A Rme2a T Kme1 Q T A Rme2a Kac S T G G K A P R K Q | H3 1-19 | R2me2a | K4me1 | R8me 2a | K9ac | free |
| J 1 | A Rme2a T Kme2 Q T A Rme2a Kac S T G G K A P R K Q | H3 1-19 | R2me2a | K4me2 | R8me 2a | K9ac | free |

| | | | | | | | |
|---|---|---|---|---|---|---|---|
| J 2 | A Rme2a T Kme3 Q T A Rme2a Kac S T G G K A P R K Q | H3 1-19 | R2me2a | K4me3 | R8me 2a | K9ac | free |
| J 3 | A Rme2a T Kac Q T A Rme2a Kac S T G G K A P R K Q | H3 1-19 | R2me2a | K4ac | R8me 2a | K9ac | free |
| J 4 | A R K S T G G K A P R K Q L A T K A A R | H3 7-26 | unmod | | | | acetylated |
| J 5 | A R K S T G G Kac A P R K Q L A T K A A R | H3 7-26 | K14ac | | | | acetylated |
| J 6 | A R K pS T G G Kac A P R K Q L A T K A A R | H3 7-26 | K14ac | S10P | | | acetylated |
| J 7 | A R K S pT G G Kac A P R K Q L A T K A A R | H3 7-26 | K14ac | T11P | | | acetylated |
| J 8 | A R K S T G G K A P Rme2s K Q L A T K A A R | H3 7-26 | R17me2 s | | | | acetylated |
| J 9 | A R K S T G G K A P Rme2a K Q L A T K A A R | H3 7-26 | R17me2 a | | | | acetylated |
| J10 | A R K S T G G K A P Cit K Q L A T K A A R | H3 7-26 | R17Cit r | | | | acetylated |
| J11 | A R K S T G G K A P R Kac Q L A T K A A R | H3 7-26 | K18ac | | | | acetylated |
| J12 | A R K S T G G Kac A P Rme2s K Q L A T K A A R | H3 7-26 | K14ac | R17me 2s | | | acetylated |
| J13 | A R K S T G G Kac A P Rme2a K Q L A T K A A R | H3 7-26 | K14ac | R17me 2a | | | acetylated |
| J14 | A R K S T G G Kac A P R Kac Q L A T K A A R | H3 7-26 | K14ac | K18ac | | | acetylated |
| J15 | A R K S T G G K A P Rme2s Kac Q L A T K A A R | H3 7-26 | R17me2 s | K18ac | | | acetylated |
| J16 | A R K S T G G K A P Rme2a Kac Q L A T K A A R | H3 7-26 | R17me2 a | K18ac | | | acetylated |
| J17 | A R K S T G G K A P Cit Kac Q L A T K A A R | H3 7-26 | R17Cit r | K18ac | | | acetylated |
| J18 | A R K S T G G Kac A P Rme2s Kac Q L A T K A A R | H3 7-26 | K14ac | R17me 2s | K18a c | | acetylated |
| J19 | A R K S T G G Kac A P Rme2a Kac Q L A T K A A R | H3 7-26 | K14ac | R17me 2a | K18a c | | acetylated |
| J20 | P R K Q L A T K A A R K S A P A T G G | H3 16-35 | unmod | | | | acetylated |
| J21 | P R K Q L A T K A A Rme2s K S A P A T G G | H3 16-35 | R26me2 s | | | | acetylated |
| J22 | P R K Q L A T K A A Rme2a K S A P A T G G | H3 16-35 | R26me2 a | | | | acetylated |
| J23 | P R K Q L A T K A A Cit K S A P A T G G | H3 16-35 | R26Cit r | | | | acetylated |

| | | | | | | | |
|---|---|---|---|---|---|---|---|
| J24 | P R K Q L A T K A A R Kme1 S A P A T G G | H3 16-35 | K27me1 | | | | acetylated |
| K 1 | P R K Q L A T K A A R Kme2 S A P A T G G | H3 16-35 | K27me2 | | | | acetylated |
| K 2 | P R K Q L A T K A A R Kme3 S A P A T G G | H3 16-35 | K27me3 | | | | acetylated |
| K 3 | P R K Q L A T K A A R Kac S A P A T G G | H3 16-35 | K27ac | | | | acetylated |
| K 4 | P R K Q L A T K A A R K pS A P A T G G | H3 16-35 | S28P | | | | acetylated |
| K 5 | P R K Q L A T K A A Rme2s Kme1 S A P A T G G | H3 16-35 | R26me2s | K27me1 | | | acetylated |
| K 6 | P R K Q L A T K A A Rme2s Kme2 S A P A T G G | H3 16-35 | R26me2s | K27me2 | | | acetylated |
| K 7 | P R K Q L A T K A A Rme2s Kme3 S A P A T G G | H3 16-35 | R26me2s | K27me3 | | | acetylated |
| K 8 | P R K Q L A T K A A Rme2s Kac S A P A T G G | H3 16-35 | R26me2s | K27ac | | | acetylated |
| K 9 | P R K Q L A T K A A Rme2s K pS A P A T G G | H3 16-35 | R26me2s | S28P | | | acetylated |
| K10 | P R K Q L A T K A A Rme2a Kme1 S A P A T G G | H3 16-35 | R26me2a | K27me1 | | | acetylated |
| K11 | P R K Q L A T K A A Rme2a Kme2 S A P A T G G | H3 16-35 | R26me2a | K27me2 | | | acetylated |
| K12 | P R K Q L A T K A A Rme2a Kme3 S A P A T G G | H3 16-35 | R26me2a | K27me3 | | | acetylated |
| K13 | P R K Q L A T K A A Rme2a Kac S A P A T G G | H3 16-35 | R26me2a | K27ac | | | acetylated |
| K14 | P R K Q L A T K A A Rme2a K pS A P A T G G | H3 16-35 | R26me2a | S28P | | | acetylated |
| K15 | P R K Q L A T K A A Cit Kme1 S A P A T G G | H3 16-35 | R26Citr | K27me1 | | | acetylated |
| K16 | P R K Q L A T K A A Cit Kme2 S A P A T G G | H3 16-35 | R26Citr | K27me2 | | | acetylated |
| K17 | P R K Q L A T K A A Cit Kme3 S A P A T G G | H3 16-35 | R26Citr | K27me3 | | | acetylated |
| K18 | P R K Q L A T K A A Cit K pS A P A T G G | H3 16-35 | R26Citr | S28P | | | acetylated |
| K19 | P R K Q L A T K A A R Kme1 pS A P A T G G | H3 16-35 | K27me1 | S28P | | | acetylated |
| K20 | P R K Q L A T K A A R Kme2 pS A P A T G G | H3 16-35 | K27me2 | S28P | | | acetylated |
| K21 | P R K Q L A T K A A R Kme3 pS A P A T G G | H3 16-35 | K27me3 | S28P | | | acetylated |

| | | | | | | | |
|---|---|---|---|---|---|---|---|
| K22 | P R K Q L A T K A A R Kac pS A P A T G G | H3 16-35 | K27ac | S28P | | | acetylated |
| K23 | P R K Q L A T K A A Rme2s Kme1 pS A P A T G G | H3 16-35 | R26me2s | K27me1 | S28P | | acetylated |
| K24 | P R K Q L A T K A A Rme2s Kme2 pS A P A T G G | H3 16-35 | R26me2s | K27me2 | S28P | | acetylated |
| L 1 | P R K Q L A T K A A Rme2s Kme3 pS A P A T G G | H3 16-35 | R26me2s | K27me3 | S28P | | acetylated |
| L 2 | P R K Q L A T K A A Rme2s Kac pS A P A T G G | H3 16-35 | R26me2s | K27ac | S28P | | acetylated |
| L 3 | P R K Q L A T K A A Rme2a Kme1 pS A P A T G G | H3 16-35 | R26me2a | K27me1 | S28P | | acetylated |
| L 4 | P R K Q L A T K A A Rme2a Kme2 pS A P A T G G | H3 16-35 | R26me2a | K27me2 | S28P | | acetylated |
| L 5 | P R K Q L A T K A A Rme2a Kme3 pS A P A T G G | H3 16-35 | R26me2a | K27me3 | S28P | | acetylated |
| L 6 | P R K Q L A T K A A Rme2a Kac pS A P A T G G | H3 16-35 | R26me2a | K27ac | S28P | | acetylated |
| L 7 | R K S A P A T G G V K K P H R Y R P G | H3 26-45 | unmod | | | | acetylated |
| L 8 | R K S A P A T G G V Kme1 K P H R Y R P G | H3 26-45 | K36me1 | | | | acetylated |
| L 9 | R K S A P A T G G V Kme2 K P H R Y R P G | H3 26-45 | K36me2 | | | | acetylated |
| L10 | R K S A P A T G G V Kme3 K P H R Y R P G | H3 26-45 | K36me3 | | | | acetylated |
| L11 | R K S A P A T G G V Kac K P H R Y R P G | H3 26-45 | K36ac | | | | acetylated |
| L12 | S G R G K G G K G L G K G G A K R H R | H4 1-19 | unmod | | | | free |
| L13 | pS G R G K G G K G L G K G G A K R H R | H4 1-19 | S1P | | | | free |
| L14 | S G Rme2s G K G G K G L G K G G A K R H R | H4 1-19 | R3me2s | | | | free |
| L15 | S G Rme2a G K G G K G L G K G G A K R H R | H4 1-19 | R3me2a | | | | free |
| L16 | S G R G Kac G G K G L G K G G A K R H R | H4 1-19 | K5ac | | | | free |
| L17 | S G R G K G G Kac G L G K G G A K R H R | H4 1-19 | K8ac | | | | free |
| L18 | S G R G K G G K G L G Kac G G A K R H R | H4 1-19 | K12ac | | | | free |
| L19 | S G R G K G G K G L G K G G A Kac R H R | H4 1-19 | K16ac | | | | free |

| | | | | | | | |
|---|---|---|---|---|---|---|---|
| L20 | pS G Rme2s G K G G K G L G K G G A K R H R | H4 1-19 | S1P | R3me2 s | | | free |
| L21 | pS G Rme2a G K G G K G L G K G G A K R H R | H4 1-19 | S1P | R3me2 a | | | free |
| L22 | pS G R G Kac G G K G L G K G G A K R H R | H4 1-19 | S1P | K5ac | | | free |
| L23 | S G Rme2s G Kac G G K G L G K G G A K R H R | H4 1-19 | R3me2s | K5ac | | | free |
| L24 | S G Rme2s G K G G Kac G L G K G G A K R H R | H4 1-19 | R3me2s | K8ac | | | free |
| M 1 | S G Rme2a G Kac G G K G L G K G G A K R H R | H4 1-19 | R3me2a | K5ac | | | free |
| M 2 | S G Rme2a G K G G Kac G L G K G G A K R H R | H4 1-19 | R3me2a | K8ac | | | free |
| M 3 | S G R G Kac G G Kac G L G K G G A K R H R | H4 1-19 | K5ac | K8ac | | | free |
| M 4 | S G R G K G G Kac G L G Kac G G A K R H R | H4 1-19 | K8ac | K12ac | | | free |
| M 5 | S G R G K G G Kac G L G K G G A Kac R H R | H4 1-19 | K8ac | K16ac | | | free |
| M 6 | S G R G K G G K G L G Kac G G A Kac R H R | H4 1-19 | K12ac | K16ac | | | free |
| M 7 | pS G Rme2s G Kac G G K G L G K G G A K R H R | H4 1-19 | S1P | R3me2 s | K5ac | | free |
| M 8 | pS G Rme2a G Kac G G K G L G K G G A K R H R | H4 1-19 | S1P | R3me2 a | K5ac | | free |
| M 9 | S G Rme2s G Kac G G Kac G L G K G G A K R H R | H4 1-19 | R3me2s | K5ac | K8ac | | free |
| M10 | S G Rme2a G Kac G G Kac G L G K G G A K R H R | H4 1-19 | R3me2a | K5ac | K8ac | | free |
| M11 | S G R G Kac G G Kac G L G Kac G G A K R H R | H4 1-19 | K5ac | K8ac | K12a c | | free |
| M12 | S G R G K G G Kac G L G Kac G G A Kac R H R | H4 1-19 | K8ac | K12ac | K16a c | | free |
| M13 | pS G Rme2s G Kac G G Kac G L G K G G A K R H R | H4 1-19 | S1P | R3me2 s | K5ac | K8ac | free |
| M14 | pS G Rme2a G Kac G G Kac G L G K G G A K R H R | H4 1-19 | S1P | R3me2 a | K5ac | K8ac | free |
| M15 | S G Rme2s G Kac G G Kac G L G Kac G G A K R H R | H4 1-19 | R3me2s | K5ac | K8ac | K12ac | free |
| M16 | S G Rme2a G Kac G G Kac G L G Kac G G A K R H R | H4 1-19 | R3me2a | K5ac | K8ac | K12ac | free |
| M17 | S G R G Kac G G Kac G L G Kac G G A Kac R H R | H4 1-19 | K5ac | K8ac | K12a c | K16ac | free |

| | | | | | | | |
|---|---|---|---|---|---|---|---|
| M18 | G K G G A K R H R K V L R D N I Q G I T | H4 11-30 | unmod | | | | acetylated |
| M19 | G Kac G G A K R H R K V L R D N I Q G I T | H4 11-30 | K12ac | | | | acetylated |
| M20 | G K G G A Kac R H R K V L R D N I Q G I T | H4 11-30 | K16ac | | | | acetylated |
| M21 | G K G G A K Rme2s H R K V L R D N I Q G I T | H4 11-30 | R17me2s | | | | acetylated |
| M22 | G K G G A K Rme2a H R K V L R D N I Q G I T | H4 11-30 | R17me2a | | | | acetylated |
| M23 | G K G G A K R H Rme2s K V L R D N I Q G I T | H4 11-30 | R19me2s | | | | acetylated |
| M24 | G K G G A K R H Rme2a K V L R D N I Q G I T | H4 11-30 | R19me2a | | | | acetylated |
| N 1 | G K G G A K R H R Kme1 V L R D N I Q G I T | H4 11-30 | K20me1 | | | | acetylated |
| N 2 | G K G G A K R H R Kme2 V L R D N I Q G I T | H4 11-30 | K20me2 | | | | acetylated |
| N 3 | G K G G A K R H R Kme3 V L R D N I Q G I T | H4 11-30 | K20me3 | | | | acetylated |
| N 4 | G K G G A K R H R Kac V L R D N I Q G I T | H4 11-30 | K20ac | | | | acetylated |
| N 5 | G K G G A K R H R K V L Rme2a D N I Q G I T | H4 11-30 | R24me2a | | | | acetylated |
| N 6 | G K G G A K R H R K V L Rme2s D N I Q G I T | H4 11-30 | R24me2s | | | | acetylated |
| N 7 | G Kac G G A Kac R H R K V L R D N I Q G I T | H4 11-30 | K12ac | K16ac | | | acetylated |
| N 8 | G K G G A Kac Rme2s H R K V L R D N I Q G I T | H4 11-30 | K16ac | R17me2s | | | acetylated |
| N 9 | G K G G A Kac Rme2a H R K V L R D N I Q G I T | H4 11-30 | K16ac | R17me2a | | | acetylated |
| N10 | G K G G A Kac R H Rme2s K V L R D N I Q G I T | H4 11-30 | K16ac | R19me2s | | | acetylated |
| N11 | G K G G A Kac R H Rme2a K V L R D N I Q G I T | H4 11-30 | K16ac | R19me2a | | | acetylated |
| N12 | G K G G A Kac R H R Kme1 V L R D N I Q G I T | H4 11-30 | K16ac | K20me1 | | | acetylated |
| N13 | G K G G A Kac R H R Kme2 V L R D N I Q G I T | H4 11-30 | K16ac | K20me2 | | | acetylated |
| N14 | G K G G A Kac R H R Kme3 V L R D N I Q G I T | H4 11-30 | K16ac | K20me3 | | | acetylated |
| N15 | G K G G A Kac R H R Kac V L R D N I Q G I T | H4 11-30 | K16ac | K20ac | | | acetylated |

| | | | | | | | |
|---|---|---|---|---|---|---|---|
| N16 | G Kac G G A Kac R H R Kme1 V L R D N I Q G I T | H4 11-30 | K12ac | K16ac | K20me1 | | acetylated |
| N17 | G Kac G G A Kac R H R Kme2 V L R D N I Q G I T | H4 11-30 | K12ac | K16ac | K20me2 | | acetylated |
| N18 | G Kac G G A Kac R H R Kme3 V L R D N I Q G I T | H4 11-30 | K12ac | K16ac | K20me3 | | acetylated |
| N19 | G Kac G G A Kac R H R Kac V L R D N I Q G I T | H4 11-30 | K12ac | K16ac | K20ac | | acetylated |
| N20 | G K G G A K R H Rme2a Kme1 V L R D N I Q G I T | H4 11-30 | R19me2a | K20me1 | | | acetylated |
| N21 | G K G G A K R H Rme2a Kme2 V L R D N I Q G I T | H4 11-30 | R19me2a | K20me2 | | | acetylated |
| N22 | G K G G A K R H Rme2a Kme3 V L R D N I Q G I T | H4 11-30 | R19me2a | K20me3 | | | acetylated |
| N23 | G K G G A K R H Rme2a Kac V L R D N I Q G I T | H4 11-30 | R19me2a | K20ac | | | acetylated |
| N24 | G K G G A K R H Rme2s Kme1 V L R D N I Q G I T | H4 11-30 | R19me2s | K20me1 | | | acetylated |
| O 1 | G K G G A K R H Rme2s Kme2 V L R D N I Q G I T | H4 11-30 | R19me2s | K20me2 | | | acetylated |
| O 2 | G K G G A K R H Rme2s Kme3 V L R D N I Q G I T | H4 11-30 | R19me2s | K20me3 | | | acetylated |
| O 3 | G K G G A K R H Rme2s Kac V L R D N I Q G I T | H4 11-30 | R19me2s | K20ac | | | acetylated |
| O 4 | G K G G A K R H R Kme1 V L Rme2a D N I Q G I T | H4 11-30 | R24me2a | K20me1 | | | acetylated |
| O 5 | G K G G A K R H R Kme2 V L Rme2a D N I Q G I T | H4 11-30 | R24me2a | K20me2 | | | acetylated |
| O 6 | G K G G A K R H R Kme3 V L Rme2a D N I Q G I T | H4 11-30 | R24me2a | K20me3 | | | acetylated |
| O 7 | G K G G A K R H R Kac V L Rme2a D N I Q G I T | H4 11-30 | R24me2a | K20ac | | | acetylated |
| O 8 | G K G G A K R H R Kme1 V L Rme2s D N I Q G I T | H4 11-30 | R24me2s | K20me1 | | | acetylated |
| O 9 | G K G G A K R H R Kme2 V L Rme2s D N I Q G I T | H4 11-30 | R24me2s | K20me2 | | | acetylated |
| O10 | G K G G A K R H R Kme3 V L Rme2s D N I Q G I T | H4 11-30 | R24me2s | K20me3 | | | acetylated |
| O11 | G K G G A K R H R Kac V L Rme2s D N I Q G I T | H4 11-30 | R24me2s | K20ac | | | acetylated |
| O12 | S G R G K Q G G K A R A K A K S R S S | H2a 1-19 | unmod | | | | free |
| O13 | pS G R G K Q G G K A R A K A K S R S S | H2a 1-19 | S1P | | | | free |

| | | | | | | | |
|---|---|---|---|---|---|---|---|
| O14 | S G R G Kac Q G G K A R A K A K S R S S | H2a 1-19 | K5ac | | | | free |
| O15 | S G R G K Q G G Kac A R A K A K S R S S | H2a 1-19 | K9ac | | | | free |
| O16 | S G R G K Q G G K A R A Kac A K S R S S | H2a 1-19 | K13ac | | | | free |
| O17 | pS G R G Kac Q G G K A R A K A K S R S S | H2a 1-19 | S1P | K5ac | | | free |
| O18 | pS G R G K Q G G Kac A R A K A K S R S S | H2a 1-19 | S1P | K9ac | | | free |
| O19 | pS G R G K Q G G K A R A Kac A K S R S S | H2a 1-19 | S1P | K13ac | | | free |
| O20 | S G R G Kac Q G G Kac A R A K A K S R S S | H2a 1-19 | K5ac | K9ac | | | free |
| O21 | S G R G Kac Q G G K A R A Kac A K S R S S | H2a 1-19 | K5ac | K13ac | | | free |
| O22 | S G R G K Q G G Kac A R A Kac A K S R S S | H2a 1-19 | K9ac | K13ac | | | free |
| O23 | pS G R G Kac Q G G Kac A R A K A K S R S S | H2a 1-19 | S1P | K5ac | K9ac | | free |
| O24 | pS G R G Kac Q G G K A R A Kac A K S R S S | H2a 1-19 | S1P | K5ac | K13a c | | free |
| P 1 | pS G R G K Q G G Kac A R A Kac A K S R S S | H2a 1-19 | S1P | K9ac | K13a c | | free |
| P 2 | S G R G Kac Q G G Kac A R A Kac A K S R S S | H2a 1-19 | K5ac | K9ac | K13a c | | free |
| P 3 | pS G R G Kac Q G G Kac A R A Kac A K S R S S | H2a 1-19 | S1P | K5ac | K9ac | K13ac | free |
| P 4 | P D P A K S A P A P K K G S K K A V T | H2B 1-19 | unmod | | | | free |
| P 5 | P D P A Kac S A P A P K K G S K K A V T | H2B 1-19 | K5ac | | | | free |
| P 6 | P D P A K S A P A P K Kac G S K K A V T | H2B 1-19 | K12ac | | | | free |
| P 7 | P D P A K S A P A P K K G pS K K A V T | H2B 1-19 | S14P | | | | free |
| P 8 | P D P A K S A P A P K K G S Kac K A V T | H2B 1-19 | K15ac | | | | free |
| P 9 | P D P A Kac S A P A P K Kac G S K K A V T | H2B 1-19 | K5ac | K12ac | | | free |
| P10 | P D P A Kac S A P A P K K G pS K K A V T | H2B 1-19 | K5ac | S14P | | | free |
| P11 | P D P A Kac S A P A P K K G S Kac K A V T | H2B 1-19 | K5ac | K15ac | | | free |

| | | | | | | | |
|---|---|---|---|---|---|---|---|
| P12 | P D P A K S A P A P K Kac G pS K K A V T | H2B 1-19 | K12ac | S14P | | | free |
| P13 | P D P A K S A P A P K Kac G S Kac K A V T | H2B 1-19 | K12ac | K15ac | | | free |
| P14 | P D P A K S A P A P K K G pS Kac K A V T | H2B 1-19 | S14P | K15ac | | | free |
| P15 | P D P A Kac S A P A P K Kac G pS K K A V T | H2B 1-19 | K5ac | K12ac | S14P | | free |
| P16 | P D P A Kac S A P A P K Kac G S Kac K A V T | H2B 1-19 | K5ac | K12ac | K15ac | | free |
| P17 | P D P A Kac S A P A P K K G pS Kac K A V T | H2B 1-19 | K5ac | S14P | K15ac | | free |
| P18 | P D P A K S A P A P K Kac G pS Kac K A V T | H2B 1-19 | K12ac | S14P | K15ac | | free |
| P19 | P D P A Kac S A P A P K Kac G pS Kac K A V T | H2B 1-19 | K5ac | K12ac | S14P | K15ac | free |
| P20 | Bio A A N W S H P Q F E K A A | Biotin, control peptide | | | | | biotinylated |
| P21 | E Q K L I S E E D L A | c-myc tag | | | | | free |
| P22 | HAc | neg. control | | | | | acetylated |
| P23 | K Kme1 Kme2 Kme3 Kac R Rme2s R Rme2a R Cit K Kme1 Kac Kme3 R K | background 01 | | | | | acetylated |
| P24 | R Rme2s K Kme1 Kac R Rme2a Kme2 K Kme3 R Kme1 Rme2s K Kac R K | background 02 | | | | | acetylated |

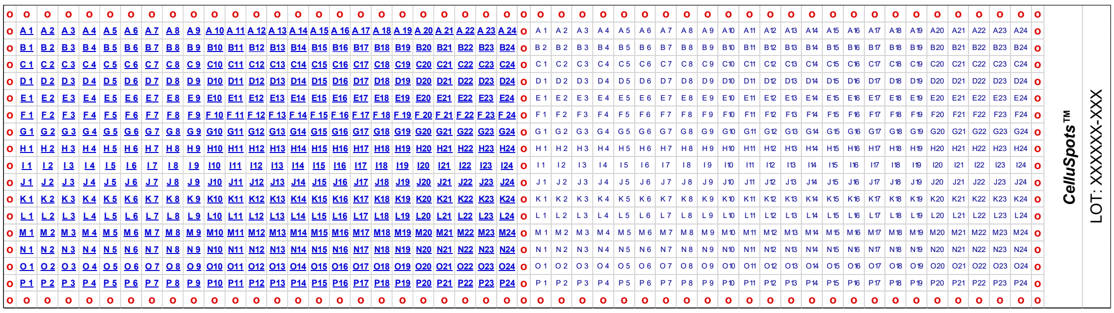

## Supplementary references


1 Young, N. L. *et al.* High throughput characterization of combinatorial histone codes. *Molecular & cellular proteomics : MCP* **8**, 2266-2284 (2009).

2 Sridharan, R. *et al.* Proteomic and genomic approaches reveal critical functions of H3K9 methylation and heterochromatin protein-1gamma in reprogramming to pluripotency. *Nature cell biology* **15**, 872-882 (2013).

3 Bock, I. *et al.* Detailed specificity analysis of antibodies binding to modified histone tails with peptide arrays. *Epigenetics* **6**, 256-263 (2011).

4 Kungulovski, G., Mauser, R., Reinhardt, R. & Jeltsch, A. Application of recombinant TAF3 PHD domain instead of anti-H3K4me3 antibody. *Epigenetics & chromatin* **9**, 11 (2016).

5 Kabsch, W. Xds. *Acta crystallographica. Section D, Biological crystallography* **66**, 125-132 (2010).

6 Evans, P. R. & Murshudov, G. N. How good are my data and what is the resolution? *Acta crystallographica. Section D, Biological crystallography* **69**, 1204-1214 (2013).

7 McCoy, A. J. *et al.* Phaser crystallographic software. *Journal of applied crystallography* **40**, 658-674 (2007).

8 Afonine, P. V. *et al.* Towards automated crystallographic structure refinement with phenix.refine. *Acta crystallographica. Section D, Biological crystallography* **68**, 352-367 (2012).

9 Minor, W., Cymborowski, M., Otwinowski, Z. & Chruszcz, M. HKL-3000: the integration of data reduction and structure solution--from diffraction images to an initial model in minutes. *Acta crystallographica. Section D, Biological crystallography* **62**, 859-866 (2006).

10 Emsley, P., Lohkamp, B., Scott, W. G. & Cowtan, K. Features and development of Coot. *Acta crystallographica. Section D, Biological crystallography* **66**, 486-501 (2010).

11 Winn, M. D. *et al.* Overview of the CCP4 suite and current developments. *Acta crystallographica. Section D, Biological crystallography* **67**, 235-242 (2011).

12 Murshudov, G. N. *et al.* REFMAC5 for the refinement of macromolecular crystal structures. *Acta crystallographica. Section D, Biological crystallography* **67**, 355-367 (2011).

13 Chen, V. B. *et al.* MolProbity: all-atom structure validation for macromolecular crystallography. *Acta crystallographica. Section D, Biological crystallography* **66**, 12-21 (2010).

14 Zucker, F., Champ, P. C. & Merritt, E. A. Validation of crystallographic models containing TLS or other descriptions of anisotropy. *Acta crystallographica. Section D, Biological crystallography* **66**, 889-900 (2010).

15 Yang, H. *et al.* Automated and accurate deposition of structures solved by X-ray diffraction to the Protein Data Bank. *Acta crystallographica. Section D, Biological crystallography* **60**, 1833-1839 (2004).

16 Adams, P. D. *et al.* PHENIX: a comprehensive Python-based system for macromolecular structure solution. *Acta crystallographica. Section D, Biological crystallography* **66**, 213-221 (2010).

17 Gildea, R. J. *et al.* iotbx.cif: a comprehensive CIF toolbox. *Journal of applied crystallography* **44**, 1259-1263 (2011).

18 Bock, I. *et al.* Application of Celluspots peptide arrays for the analysis of the binding specificity of epigenetic reading domains to modified histone tails. *BMC biochemistry* **12**, 48 (2011).
